# Major Bioactive Compounds in Seeds, Husks, and Leaves of Selected Genotypes of *Coffea canephora* cv. Conilon from Three Consecutive Crops

**DOI:** 10.3390/plants14071040

**Published:** 2025-03-27

**Authors:** Juliana DePaula, Fábio Luiz Partelli, Alessandro M. Batista, Veronica Calado, Adriana Farah

**Affiliations:** 1 Food Chemistry and Bioactivity Laboratory & Coffee Research Core—NUPECAFÉ, Nutrition Institute, Federal University of Rio de Janeiro (UFRJ), Ilha do Fundão, CCS Bloco J, Rio de Janeiro 21941-902, RJ, Brazil; julianadepaula@nutricao.ufrj.br (J.D.); alessandromaiabatista@gmail.com (A.M.B.); 2 Departamento de Ciências Agrárias e Biológicas, Centro Universitário do Norte do Espírito Santo, Universidade Federal do Espírito Santo, São Mateus 29932-900, ES, Brazil; partelli@yahoo.com.br; 3 Thermo Analysis and Rheology Laboratory, Chemistry School, Federal University of Rio de Janeiro (UFRJ), Ilha do Fundão, CT Bloco K, Rio de Janeiro 21941-972, RJ, Brazil; calado@eq.ufrj.br

**Keywords:** robusta coffee, canefora coffee, coffee seed, coffee husk, coffee leaf, chlorogenic acids, caffeine, trigonelline

## Abstract

This study aimed to investigate: (1) the bioactive profile of seeds, husks, and leaves of selected conilon coffee genotypes (*n* = 42) from three consecutive crops for the selection of plants to meet health interests, (2) the variability in the content of these bioactive compounds over the crops, and (3) possible correlations among the contents of the evaluated compounds in the different parts of the plant. Selected conilon plants were reproduced by clonal propagation. Bioactive compounds were analyzed using HPLC-DAD. Eight chlorogenic acids (CGA), caffeine, trigonelline, and minor phenolic compounds were quantified (dry basis) in all extracts. CGA contents in seeds, husks, and leaves ranged between 3.71 and 9.71 g/100 g, 0.43 and 1.65 g/100 g, and 0.80 and 2.22 g/100 g, respectively. Caffeine contents ranged between 1.21 and 2.63 g/100 g, 0.13 and 0.84 g/100 g, and 0.33 and 2.01 g/100 g in seeds, husks, and leaves, respectively. Trigonelline contents ranged between 0.83 and 1.12 g/100 g, 0.59 and 1.24 g/100 g, and 0.74 and 1.84 g/100 g, respectively. Variation among the three crops was observed to be higher for CGA. A discrete correlation between CGA and caffeine was observed in the seeds (r: 0.72, *p* = 0.003). Some of the genotypes showed consistently higher contents of these bioactive compounds than others (not only in the seeds but also in the husks and leaves), being good candidates for cultivar registration to meet various market demands in the food and pharmaceutical industries. Studies that evaluate the potential use of new genotypes and byproducts are important for diversification and maximum use of coffee plants, promoting sustainability and financial return to the farmers and the producing country.

## 1. Introduction

In the last few decades, epidemiological studies have reported several health benefits of regular coffee drinking, such as reduced risk of cardiovascular disease, diabetes, multiple cancers, and all-cause mortality, leading to its inclusion in the hall of functional foods [1]. This contributed to a worldwide increase in consumption, reaching 10.7 million tons in 2024 [2]. Most recently, for the same reasons, the unsweetened beverage was qualified to bear the “healthy” claim by the American Food and Drug Administration [3]. Even though most health-related benefits of coffee drinking have been attributed to the beverage as such and not to individual compounds, mechanistic studies have suggested that specific bioactive compounds such as chlorogenic acids (CGA), caffeine, and trigonelline play key roles as co-adjuvant agents in disease prevention because of their antioxidant and anti-inflammatory activities [1].

Among over a hundred and thirty coffee species identified to date, two were spread for cultivation around the world’s tropical regions and have important market value, *Coffea arabica* and *Coffea canephora*. Canefora seeds (commercially represented mainly by the robusta and conilon cultivars) typically produce beverages with lower complexity than arabica beverages, low acidity and neutral or cereal sensory characteristics, and high contents of soluble solids, including CGA and caffeine, which can be interesting regarding health [4]. Historically, there has been a disparity in the production and marketing of arabica and canefora species due to the different acceptability of beverages. Despite the richer aroma and greater appreciation of arabica seeds, canefora seeds show important properties in the ground roasted segment, being widely blended with arabica seeds to increase body and highlight arabica’s flavor [4]. It also increases yield in the soluble segment. Currently, with the development of innovative post-harvest methodologies, there has been continuously growing interest in canefora seeds because of their flavor improvement, especially those gone through fermentative processes, which can lead to notes resembling cocoa nibs, or exquisite liquors and other natural and pleasant flavors. This, along with other qualities, has allowed canefora seeds to be included among the specialty coffees and gain market value. Furthermore, because the canefora plants tend to be more resistant to higher temperatures than arabica, its cultivation is promising in arabica-producing regions affected by climate variations as the result of global warming.

Although health improvement is not historically the main reason why people drink coffee, the higher demand for canefora seeds in the market propelled breeders to search for and develop new cultivars not only aiming for higher productivity, higher resistance to climate changes and pests, and for good cup quality, but also for higher contents of soluble solids and bioactive compounds [1,4,5].

Brazil is the largest world coffee producer and exporter of arabica coffee and the second producer of canefora coffee, mainly the conilon cultivar, accounting for about 28% of the world’s canefora production [6]. In the last three decades, the yield of canefora coffee has increased remarkably in the country, with Espírito Santo, Rondônia, and Bahia states producing about 96% of the total canefora production [7]. As a side effect of coffee production, a large amount of fruit husks and leaves are produced yearly, and as the seed’s productivity increases, so does the generation of these byproducts [8,9]. It has been estimated in the last decade that 3.3 tons of leaves per hectare were discarded during coffee harvest in Brazil [10]. Leaf loss is lower during manual harvesting, although it still exists. Leaves are also discarded during the pruning of the trees. Because coffee husks and leaves are sources of caffeine, CGA and several additional phenolic and non-phenolic bioactive compounds, if inappropriately discarded, the wasted husks and leaves may cause environmental problems such as unfavorable alteration of the soil microbiota due to the antimicrobial effect of these bioactive compounds and the arrival of solid waste in rivers and underground waters [11,12].

Ethnomedicinal infusions made from husks and leaves have been expressively used for centuries in countries like Ethiopia, Yemen, and Bolívia. Infusions from sun-dried arabica leaves have been used since the early 19th century for the treatment of intestinal disorders, AIDS complications, and tuberculosis [13,14,15]. These properties demonstrate the promising potential of using coffee leaves and husks to produce new beverages and blended formulations or as alternative matrices for extracting bioactive compounds that can be used in the food and drug industry. Despite the expressive coffee production worldwide and the large production of byproducts, studies investigating the bioactive composition of the leaves and husks are still scarce, especially those from canefora species.

For the aforementioned reasons, the aims of this study were to investigate the bioactive profile of promising seeds, husks, and leaves of canefora genotypes from consecutive crops for selection of genotypes to meet health interests, to investigate the variability in the content of bioactive compounds over the consecutive crops, and to investigate possible correlations among the contents of the evaluated compounds in the different parts of the plant.

## 2. Results and Discussion

### 2.1. Water Content

On average, after drying, considering the three crops, seeds, husks, and leaves presented 8.7%, 11.2%, and 10.4% of water, respectively (Table 1). These values were similar in all three crops because the material was dried in the same conditions. The water contents were used to express the contents of bioactive compounds on a dry basis (db).

### 2.2. Soluble Solids Contents of Green Seeds

Table 2 presents the content of soluble solids in the green seeds evaluated in the study. Considering the three crops, the contents ranged between 2.9 and 5.0 °Brix. While most genotypes showed very small crop variability (Coefficient of Variation − CV% < 2%), seven of them showed CV% between 7% and 8%. As aforementioned, the selection of cultivars with high soluble solids is of great value to the ground roasted and soluble coffee industry [16]. It is worth noting that soluble solids contents can vary according to the degree of roasting (which is not the case in this study) and particle size (grinding). In Table 2, some genotypes (in bold) can be highlighted because of their consistently high soluble solids content (4.2–4.7 °Brix, CV < 7%) in all evaluated crops, about 10 to 20% higher than the remaining genotypes. The potential for using these genotypes in the soluble coffee industry will be explored later in this report.

### 2.3. Bioactive Compounds in Green Seeds, Husks, and Leaves

#### 2.3.1. Chlorogenic Acids and Flavonoids

Table 3a–c present total CGA contents in seeds, husks, and leaves of the evaluated C. canephora genotypes from three consecutive crops. Eight CGA compounds were quantified in all samples of seeds, husks, and leaves: 3-CQA, 4-CQA, 5-CQA, 4-FQA, 5-FQA, 3,4-diCQA, 3,5-diCQA and 4,5-diCQA. An additional CGA, 3-FQA isomer, was identified only in the seeds. Rutin was quantified in the husks and leaves.

CGA are the main phenolic compounds in the coffee plant’s seeds and other parts [17,18]. Considering the three crops evaluated, their total contents in seeds varied from 3.71 to 9.71 g/100 g db. These contents are in accordance with literature reports for conilon and robusta coffees (4–9 g/100 g db) [19]. It is known that CGA contents in green coffee seeds can vary widely according to genetics, physiological conditions, including degree of fruit maturation [17], edaphoclimatic conditions, and agricultural practices [20,21,22], for example, zinc fertilization increased CGA contents in arabica coffee [23]. Also, in a study by Monteiro and Farah [20], high variability in the CGA content in arabica cultivars was observed among different crops, and therefore, the authors did not recommend choosing cultivars based on the CGA contents of a single crop and suggested that ideally, at least three consecutive crops should be evaluated.

It has been generally considered that CGA are stored forms of cinnamic acid derivatives and precursors for the lignin biosynthetic pathway in plants [24] and that this may be their role during the germination of coffee seeds [25]. It has also been speculated that CGA function as chemical defence compounds to protect against assorted pests and pathogens since they have antioxidant and antibiotic properties [17]. Phenolic compounds have been identified as the most common allelochemicals produced by higher plants [25]. CQA and di-CQA are closely associated with chloroplasts in very young leaves, and this association suggests that these compounds have a protective role against light damage [26]. They may also be involved in the response to different abiotic stresses such as drought and temperature [27,28].

Regarding the distribution of CGA classes and compounds in the seeds, CQA were the most abundant compounds, as expected, representing about 70% of total CGA, with 5-CQA being the main isomer (67% of total CQA). FQA were the second most abundant CGA class, accounting for about 17% of total CGA. 5-FQA corresponded to approximately 88% of FQA isomers. Total diCQA represented about 13% of total CGA in the seeds, with 3,5-diCQA corresponding to 55% of total diCQA, followed by 4,5-diCQA and 3,4-diCQA.

Outstanding mean contents (8.40–9.27 g/100 g db) were observed in eight seed genotypes (Table 3a, in bold), compared to those described in the literature for canefora coffees [19]. These values were about 30% higher than the mean value for the remaining genotypes. Their lower CV% among crops indicate consistency in the chemical composition of these genotypes over the years.

In husks (Table 3b), contents ranged between 0.43 and 1.65 g/100 g db, and in leaves (Table 3c), 0.80 and 2.22 g/100 g db. Generally, the CGA contents in the seeds were 87% and 80% higher (*p* = 0.001) than those observed in the dried husks and leaves, respectively, showing concentration in seeds. De Almeida et al. [29] also reported a significant variation in the contents of 5-CQA, 3,4-diCQA, 3,5-diCQA, and 4,5-diCQA in three samples of C. arabica leaves from three regions of Brazil (0.7 to 2.5 g/100 g db). Similar contents of total CGA to those observed in this study were reported for robusta coffee leaves from Uganda (1.9–2.2 g/100 g db) [18]. No scientific data on CGA content in C. canephora husks was found for comparison purposes. Regarding outstanding mean values for CGA in husks, ten genotypes contained 1.16 to 1.54 g/100 g db, 200% to 300% higher than those observed in the remaining genotypes (Table 3b). In the leaves, twelve genotypes presented outstanding mean values, between 1.59 and 2.05 g/100 g db, about 30% higher than the remaining genotypes (Table 3c). The genotype Cheique stood out about total CGA mean contents in seeds, husks, and leaves in the three crops evaluated. This genotype is of great interest, given that all parts of the plant can be sources of the substance for several purposes, including the farmaceutical industry.

Regarding the CGA compounds distribution in coffee husks and leaves, CQA represented about 65% and 72% of the total CGA, respectively, with 5-CQA representing 64% and 75% of the total CQA, respectively. DiCQA was the second most abundant group of compounds, accounting for about 25% and 20% of total CGA in coffee husks and leaves, respectively, with 3,5-diCQA representing about 40% and 48% of the total diCQA, respectively. FQA represented about 10% and 8% of total CGA in the husk and leaves, respectively. The predominance of 5-CQA and 3,5-diCQA isomers in leaves of the genus Coffea has been consistently reported in the literature [18,26,29,30], as well as the absence of the 3-FQA isomer in the husks [18,30]—which was confirmed in the present study.

Regarding the variability over the three crops, the CV% ranged from 1.1–28.7% in seeds, 1.2–39.1% in husks, and 5.3–42.7% in leaves. The large variation found for a few genotypes was probably caused by edaphoclimatic conditions [20,31] and due to plant stress conditions, such as the need for protection against UV rays, pests, and mechanical injury [17]. Monteiro and Farah [20] also observed fluctuating CGA content in three to five consecutive crops.

As expected, no CGA lactones were identified in the samples of seeds, husks, and leaves investigated due to the low temperatures used in the drying process (lower than 50 °C). High drying temperatures have previously led to CGA lactones formation in roasted coffee seeds [32] and toasted maté leaves [33].

Regarding flavonoids, considering the three crops evaluated, the husks presented rutin contents between 58.68 and 311.71 mg/100 g db. Similar contents were reported for arabica coffee husk from Nicaragua (90 mg/100 g db) [34]. In this study, the leaves presented rutin contents between 43.44 and 103.45 mg/100 g db. Higher contents were reported for arabica coffee leaves from Brazil (811 mg/100 g db) [29] and green maté leaves (559 mg/100 g db) [35]. Quercetin and kaempferol were not identified in the seeds, husks, and leaves investigated. No scientific data on the contents of rutin or other flavonoids in conilon husks and leaves were found for comparison.

**Table 3:** Total CGA contents (g/100 g) in seeds (**a**), husks (**b**) and leaves (**c**) of selected *C. canephora* cv. conilon genotypes, from three consecutive crops.

(**a**)											
**Genotype**	***Crop 1***	***Crop 2***	***Crop 3***	Mean *	***CV (%)***	**Genotype**	***Crop 1***	***Crop 2***	***Crop 3***	Mean *	***CV (%)***
Verdim R	6.66 ± 0.03 ^a^	6.18 ± 0.08 ^b^	5.85 ± 0.03 ^c^	6.23 ± 0.40	6.48	Z38	6.80 ± 0.05 ^b^	6.42 ± 0.05 ^c^	7.06 ± 0.10 ^a^	6.76 ± 0.32	4.78
B01	7.02 ± 0.04 ^c^	8.77 ± 0.10 ^a^	7.87 ± 0.02 ^b^	7.89 ± 0.87	11.06	Z18	6.90 ± 0.01 ^b^	7.41 ± 0.06 ^a^	6.91 ± 0.07 ^b^	7.07 ± 0.29	4.12
Bicudo	7.39 ± 0.04 ^a^	8.32 ± 0.08 ^b^	8.20 ± 0.01 ^b c^	7.97 ± 0.50	6.33	**Z17**	**8.60 ± 0.02 ^c^**	**9.16 ± 0.08 ^a^**	**8.83 ± 0.08 ^b^**	**8.86 ± 0.28**	**3.17**
Alecrim	7.09 ± 0.04 ^c^	7.75 ± 0.05 ^b^	8.28 ± 0.04 ^a^	7.71 ± 0.60	7.75	Z21	5.30 ± 0.02 ^c^	5.85 ± 0.02 ^b^	6.75 ± 0.02 ^a^	5.97 ± 0.74	12.32
700	6.33 ± 0.05 ^c^	6.81 ± 0.13 ^b^	7.85 ± 0.05 ^a^	7.00 ± 0.77	11.08	Z36	7.07 ± 0.05 ^b^	7.94 ± 0.05 ^a^	7.85 ± 0.04 ^a^	7.62 ± 0.48	6.33
CH1	5.22 ± 0.02 ^c^	6.06 ± 0.06 ^b^	7.94 ± 0.03 ^a^	6.41 ± 1.39	21.72	Ouro negro	7.60 ± 0.04 ^c^	8.54 ± 0.09 ^a^	8.09 ± 0.04 ^b^	8.08 ± 0.47	5.81
Imbigudinho	5.72 ± 0.01 ^c^	6.24 ± 0.03 ^b^	7.51 ± 0.08 ^a^	6.49 ± 0.92	14.15	18	5.51 ± 0.04 ^c^	6.45 ± 0.06 ^b^	7.70 ± 0.02 ^a^	6.55 ± 1.10	16.75
AT	5.01 ± 0.04 ^c^	6.03 ± 0.05 ^b^	8.59 ± 0.04 ^a^	6.55 ± 1.85	28.20	**Tardio C**	**8.62 ± 0.02 ^b^**	**9.09 ± 0.01 ^a^**	**9.16 ± 0.08 ^a^**	**8.96 ± 0.29**	**3.27**
**Graudão HP**	**8.88 ± 0.05 ^c^**	**9.71 ± 0.06 ^a^**	**9.22 ± 0.08 ^b^**	**9.27 ± 0.42**	**4.50**	A1	7.56 ± 0.03 ^a^	6.46 ± 0.03 ^c^	7.22 ± 0.03 ^b^	7.08 ± 0.56	7.96
Valcir P	6.73 ± 0.04 ^b^	8.22 ± 0.11 ^a^	8.12 ± 0.07 ^a^	7.69 ± 0.83	10.82	**Cheique**	**8.05 ± 0.03 ^c^**	**8.29 ± 0.02 ^b^**	**9.14 ± 0.02 ^a^**	**8.49 ± 0.57**	**6.72**
Beira Rio 8	6.70 ± 0.04 ^c^	8.82 ± 0.04 ^b^	8.95 ± 0.05 ^a^	8.16 ± 1.26	15.47	P2	6.61 ± 0.07 ^b^	7.97 ± 0.01 ^a^	7.89 ± 0.03 ^a^	7.49 ± 0.76	10.15
**Tardio V**	**8.15 ± 0.03 ^b^**	**9.50 ± 0.14 ^a^**	**8.29 ± 0.03 ^b^**	**8.65 ± 0.74**	**8.59**	**Emcapa 02**	**8.72 ± 0.06 ^c^**	**9.39 ± 0.04 ^a^**	**9.11 ± 0.06 ^b^**	**9.07 ± 0.34**	**3.73**
AP	6.43 ± 0.03 ^b^	7.53 ± 0.08 ^a^	7.40 ± 0.04 ^a^	7.12 ± 0.60	8.43	Emcapa 153	6.26 ± 0.03 ^b^	5.95 ± 0.04 ^c^	7.10 ± 0.07 ^a^	6.44 ± 0.59	9.18
L80	3.71 ± 0.04 ^c^	3.93 ± 0.03 ^b^	4.75 ± 0.05 ^a^	4.13 ± 0.05	13.29	P1	7.11 ± 0.01 ^a^	5.99 ± 0.05 ^b^	7.03 ± 0.04 ^a^	6.71 ± 0.62	9.27
Bamburral	6.05 ± 0.04 ^c^	6.74 ± 0.04 ^b^	7.54 ± 0.01 ^a^	6.77 ± 0.75	11.00	LB1	6.93 ± 0.02 ^b^	6.20 ± 0.02 ^c^	7.13 ± 0.08 ^a^	6.75 ± 0.49	7.22
Pirata	5.25 ± 0.04 ^c^	6.84 ± 0.09 ^b^	7.17 ± 0.02 ^a^	6.42 ± 1.03	15.98	**122**	**8.49 ± 0.03 ^a^**	**8.55 ± 0.03 ^a^**	**8.37 ± 0.06 ^b^**	**8.47 ± 0.09**	**1.08**
Peneirão	7.36 ± 0.02 ^c^	8.26 ± 0.06 ^a^	7.73 ± 0.03 ^b^	7.78 ± 0.45	5.81	**Verdim D**	**8.14 ± 0.01 ^c^**	**8.47 ± 0.03 ^b^**	**8.58 ± 0.05 ^a^**	**8.40 ± 0.23**	**2.70**
Z39	4.34 ± 0.05 ^c^	5.65 ± 0.03 ^b^	5.93 ± 0.07 ^a^	5.31 ± 0.85	15.96	Emcapa 143	6.06 ± 0.05 ^c^	6.40 ± 0.06 ^b^	6.81 ± 0.04 ^a^	6.42 ± 0.38	5.84
Z35	7.91 ± 0.05 ^b^	8.67 ± 0.02 ^a^	7.83 ± 0.04 ^b^	8.13 ± 0.46	5.69	Ouro negro 1	6.85 ± 0.04 ^b^	7.21 ± 0.03 ^a^	6.90 ± 0.03 ^b^	6.99 ± 0.20	2.79
Z40	5.99 ± 0.04 ^c^	7.42 ± 0.07 ^b^	7.77 ± 0.01 ^a^	7.06 ± 0.94	13.37	Ouro negro 2	7.49 ± 0.07 ^b^	7.36 ± 0.05 ^c^	8.55 ± 0.02 ^a^	7.80 ± 0.65	8.39
Z29	7.17 ± 0.05 ^a^	6.15 ± 0.14 ^b^	7.02 ± 0.02 ^c^	6.78 ± 0.05	8.14	Clementino	6.22 ± 0.08 ^c^	6.67 ± 0.05 ^b^	7.01 ± 0.05 ^a^	6.64 ± 0.40	5.95
Note (**a**): * Average total CGA contents in the three consecutive crops (2018, 2019, and 2020). Total CGA: sum of CQA + FQA + diCQA. CQA: sum of 3-CQA (3-caffeoylquinic acid) + 4-CQA (4-caffeoylquinic acid) + 5-CQA (5-caffeoylquinic acid). FQA sum of 3-FQA (3-feruloylquinic acid) + 4-FQA (4-feruloylquinic acid) + 5-FQA (5-feruloylquinic acid). diCQA: sum of 3,4-diCQA (3,4-dicaffeoylquinic acid) + 3,5-diCQA (3,5-dicaffeoylquinic acid) + 4,5-diCQA (4,5-dicaffeoylquinic acid). CV: coefficient of variation. Different letters for the same genotype indicate statistical differences between crops by ANOVA (*p* < 0.05). Values in bold indicate genotypes that stood out for consistently high contents of total CGA (>8%, CV < 9%), compared to average contents described in the literature [19] and with the remaining genotypes.											
(**b**)											
**Genotype**	***Crop 1***	***Crop 2***	***Crop 3***	Mean *	***CV (%)***	**Genotype**	***Crop 1***	***Crop 2***	***Crop 3***	Mean *	***CV (%)***
Verdim R	0.72 ± 0.02 ^b^	0.95 ± 0.05 ^a^	1.01 ± 0.02 ^a^	0.89 ± 0.15	17.43	Z38	0.48 ± 0.03 ^c^	0.55 ± 0.04 ^b^	0.67 ± 0.02 ^a^	0.57 ± 0.09	16.68
B01	0.97 ± 0.05 ^a^	0.85 ± 0.03 ^b^	0.80 ± 0.05 ^b^	0.87 ± 0.09	10.08	Z18	0.43 ± 0.01 ^b^	0.52 ± 0.05 ^a^	0.48 ± 0.03 ^b^	0.48 ± 0.04	9.30
Bicudo	0.79 ± 0.02 ^a^	0.77 ± 0.03 ^a^	0.68 ± 0.02 ^b^	0.75 ± 0.05	7.20	Z17	0.65 ± 0.01 ^b^	0.92 ± 0.07 ^a^	0.91 ± 0.01 ^a^	0.83 ± 0.16	18.79
**Alecrim**	**1.16 ± 0.03^b^**	**1.37 ± 0.05 ^a^**	**1.38 ± 0.01 ^a^**	**1.30 ± 0.12**	**9.46**	Z21	0.65 ± 0.02 ^c^	0.75 ± 0.07 ^b^	0.82 ± 0.05 ^a^	0.74 ± 0.09	11.63
**700**	**1.06 ± 0.02 ^b^**	**1.30 ± 0.03 ^a^**	**1.12 ± 0.05 ^b^**	**1.16 ± 0.03**	**10.82**	Z36	0.83 ± 0.04 ^c^	0.96 ± 0.04 ^b^	1.27 ± 0.05 ^a^	1.02 ± 0.22	21.83
CH1	0.68 ± 0.03 ^b^	0.98 ± 0.04 ^a^	1.05 ± 0.02 ^a^	0.91 ± 0.20	21.72	Ouro negro	0.71 ± 0.02 ^c^	0.86 ± 0.04 ^a^	0.84 ± 0.03 ^b^	0.80 ± 0.08	9.73
Imbigudinho	0.58 ± 0.01 ^b^	0.71 ± 0.03 ^a^	0.68 ± 0.04 ^a^	0.66 ± 0.07	10.34	18	0.66 ± 0.00 ^c^	0.96 ± 0.02 ^b^	1.07 ± 0.01 ^a^	0.90 ± 0.22	24.10
**AT**	**1.03 ± 0.03 ^c^**	**1.17 ± 0.04 ^b^**	**1.48 ± 0.02 ^a^**	**1.23 ± 0.23**	**18.78**	**Tardio C**	**1.51 ± 0.01 ^b^**	**1.65 ± 0.04 ^a^**	**1.45 ± 0.03 ^c^**	**1.54 ± 0.10**	**6.67**
Graudão HP	0.53 ± 0.01 ^c^	0.81 ± 0.05 ^b^	1.27 ± 0.02 ^a^	0.87 ± 0.09	42.47	A1	0.80 ± 0.02 ^b^	1.02 ± 0.06 ^a^	1.06 ± 0.05 ^a^	0.96 ± 0.14	14.46
Valcir P	0.93 ± 0.04 ^b^	1.00 ± 0.03 ^b^	1.32 ± 0.01 ^a^	1.08 ± 0.21	19.34	**Cheique**	**1.43 ± 0.04 ^b^**	**1.59 ± 0.02 ^a^**	**1.27 ± 0.02 ^c^**	**1.43 ± 0.16**	**10.95**
Beira Rio 8	0.50 ± 0.01 ^b^	0.82 ± 0.06 ^a^	0.88 ± 0.03 ^a^	0.73 ± 0.20	27.80	**P2**	**1.54 ± 0.02 ^a^**	**1.39 ± 0.05 ^b^**	**1.24 ± 0.03 ^c^**	**1.39 ± 0.15**	**10.65**
**Tardio V**	**1.05 ± 0.02 ^c^**	**1.26 ± 0.01 ^a^**	**1.15 ± 0.04 ^b^**	**1.16 ± 0.10**	**9.04**	Emcapa 02	0.59 ± 0.03 ^b^	0.83 ± 0.03 ^a^	0.88 ± 0.01 ^a^	0.77 ± 0.15	19.99
AP	0.91 ± 0.01 ^c^	1.25 ± 0.00 ^a^	1.19 ± 0.03 ^b^	1.12 ± 0.18	15.99	**Emcapa 153**	**1.01 ± 0.02 ^c^**	**1.25 ± 0.05 ^b^**	**1.37 ± 0.03 ^a^**	**1.21 ± 0.18**	**14.84**
L80	0.69 ± 0.01 ^c^	0.82 ± 0.01 ^b^	0.95 ± 0.03 ^a^	0.82 ± 0.13	15.73	**P1**	**1.05 ± 0.01 ^c^**	**1.26 ± 0.02 ^b^**	**1.37 ± 0.01 ^a^**	**1.23 ± 0.16**	**13.36**
Bamburral	0.98 ± 0.02 ^c^	1.13 ± 0.03 ^a^	1.05 ± 0.01 ^b^	1.06 ± 0.07	6.94	LB1	0.70 ± 0.02 ^a^	0.59 ± 0.06 ^b^	0.59 ± 0.03 ^b^	0.63 ± 0.06	9.95
Pirata	0.62 ± 0.01 ^b^	0.55 ± 0.06 ^b^	0.71 ± 0.03 ^a^	0.63 ± 0.08	12.31	122	0.58 ± 0.02 ^c^	0.74 ± 0.02 ^b^	0.81 ± 0.02 ^a^	0.71 ± 0.12	17.02
Peneirão	0.60 ± 0.02 ^b^	0.62 ± 0.05 ^a b^	0.71 ± 0.04 ^a^	0.64 ± 0.06	9.14	Verdim D	0.64 ± 0.02 ^c^	0.93 ± 0.04 ^b^	1.03 ± 0.01 ^a^	0.87 ± 0.20	23.18
Z39	0.57 ± 0.02 ^b^	0.48 ± 0.03 ^c^	0.69 ± 0.04 ^a^	0.58 ± 0.11	18.42	Emcapa 143	0.79 ± 0.02 ^b^	1.07 ± 0.04 ^a^	1.03 ± 0.04 ^a^	0.96 ± 0.15	16.05
Z35	0.46 ± 0.00 ^b^	0.47 ± 0.04 ^b^	0.55 ± 0.03 ^a^	0.50 ± 0.05	10.13	**Ouro negro 1**	**1.10 ± 0.01 ^b^**	**1.51 ± 0.05 ^a^**	**1.49 ± 0.03 ^a^**	**1.37 ± 0.23**	**16.86**
Z40	0.71 ± 0.02 ^a^	0.72 ± 0.05 ^a^	0.78 ± 0.04 ^a^	0.74 ± 0.04	5.29	Ouro negro 2	0.99 ± 0.03 ^b^	1.22 ± 0.01 ^a^	1.24 ± 0.02 ^a^	1.15 ± 0.14	11.81
Z29	0.66 ± 0.02 ^b^	0.80 ± 0.03 ^a^	0.67 ± 0.05 ^b^	0.71 ± 0.08	11.31	Clementino	0.94 ± 0.02 ^c^	1.23 ± 0.03 ^b^	1.32 ± 0.03 ^a^	1.16 ± 0.20	17.82
Note (**b**): * Average total CGA contents of the three consecutive crops (2018, 2019, and 2020). Total CGA: sum of CQA + FQA + diCQA. CQA: sum of 3-CQA (3-caffeoylquinic acid) + 4-CQA (4-caffeoylquinic acid) + 5-CQA (5-caffeoylquinic acid). FQA: sum of 4-FQA (4-feruloylquinic acid) + 5-FQA (5-feruloylquinic acid). diCQA: sum of 3,4-diCQA (3,4-dicaffeoylquinic acid) + 3,5-diCQA (3,5-dicaffeoylquinic acid) + 4,5-diCQA (4,5-dicaffeoylquinic acid). CV: coefficient of variation. Different letters for the same genotype indicate statistical differences between crops by ANOVA (*p* < 0.05). Values in bold were consistently outstanding compared to other genotypes.											
(**c**)											
**Genotype**	***Crop 1***	***Crop 2***	***Crop 3***	Mean *	***CV (%)***	**Genotype**	***Crop 1***	***Crop 2***	***Crop 3***	Mean *	***CV (%)***
Verdim R	1.57 ± 0.01 ^a^	1.44 ± 0.03 ^b^	1.43 ± 0.03 ^b^	1.48 ± 0.08	5.18	Z38	1.15 ± 0.02 ^c^	1.26 ± 0.01 ^b^	1.38 ± 0.02 ^a^	1.26 ± 0.11	9.09
B01	0.97 ± 0.01 ^b^	0.91 ± 0.01 ^c^	1.20 ± 0.03 ^a^	1.03 ± 0.15	14.81	**Z18**	**1.57 ± 0.02 ^c^**	**1.73 ± 0.02 ^b^**	**1.86 ± 0.02 ^a^**	**1.72 ± 0.15**	**8.46**
Bicudo	1.31 ± 0.01 ^b^	1.31 ± 0.03 ^b^	1.50 ± 0.02 ^a^	1.37 ± 0.11	7.74	Z17	1.25 ± 0.03 ^b^	1.27 ± 0.07 ^b^	1.56 ± 0.03 ^a^	1.36 ± 0.17	12.74
**Alecrim**	**1.56 ± 0.01 ^b^**	**1.59 ± 0.07 ^b^**	**1.86 ± 0.02 ^a^**	**1.67 ± 0.16**	**9.71**	Z21	1.80 ± 0.02 ^a^	1.47 ± 0.06 ^b^	1.18 ± 0.07 ^c^	1.48 ± 0.31	20.97
**700**	**1.70 ± 0.02 ^c^**	**1.81 ± 0.03 ^b^**	**1.94 ± 0.02 ^a^**	**1.82 ± 0.12**	**6.67**	Z36	1.24 ± 0.02 ^b^	1.27 ± 0.02 ^b^	1.34 ± 0.03 ^a^	1.28 ± 0.05	4.08
CH1	1.01 ± 0.02 ^c^	1.16 ± 0.03 ^b^	1.40 ± 0.03 ^a^	1.19 ± 0.20	16.49	Ouro negro	1.24 ± 0.02 ^a^	1.09 ± 0.04 ^b^	1.27 ± 0.02 ^a^	1.20 ± 0.10	8.13
Imbigudinho	1.16 ± 0.00 ^b^	1.09 ± 0.05 ^c^	1.30 ± 0.02 ^a^	1.18 ± 0.11	9.01	18	0.97 ± 0.02 ^b^	0.84 ± 0.03 ^c^	1.20 ± 0.02 ^a^	1.00 ± 0.18	18.09
AT	1.15 ± 0.01 ^b^	1.27 ± 0.03 ^a^	1.16 ± 0.04 ^b^	1.19 ± 0.07	5.85	Tardio C	0.99 ± 0.03 ^c^	1.06 ± 0.00 ^b^	1.25 ± 0.02 ^a^	1.10 ± 0.13	12.22
Graudão HP	1.03 ± 0.02 ^c^	1.13 ± 0.02 ^b^	1.22 ± 0.02 ^a^	1.13 ± 0.10	8.50	**A1**	**1.90 ± 0.02 ^b^**	**1.91 ± 0.03 ^b^**	**2.00 ± 0.06 ^a^**	**1.94 ± 0.06**	**2.89**
**Valcir P**	**1.97 ± 0.02 ^c^**	**2.13 ± 0.03 ^b^**	**2.05 ± 0.02 ^b^**	**2.05 ± 0.08**	**3.73**	**Cheique**	**1.53 ± 0.01 ^b^**	**1.54 ± 0.02 ^b^**	**1.69 ± 0.02 ^a^**	**1.59 ± 0.09**	**5.70**
**Beira Rio 8**	**1.54 ± 0.03 ^c^**	**1.68 ± 0.02 ^b^**	**1.78 ± 0.01 ^a^**	**1.67 ± 0.12**	**7.00**	P2	1.67 ± 0.02 ^a^	1.73 ± 0.03 ^a^	1.38 ± 0.03 ^b^	1.59 ± 0.19	11.90
Tardio V	0.87 ± 0.02 ^c^	1.04 ± 0.02 ^b^	1.26 ± 0.05 ^a^	1.06 ± 0.20	18.70	**Emcapa 02**	**1.83 ± 0.01 ^b^**	**2.22 ± 0.04 ^a^**	**1.80 ± 0.03 ^b^**	**1.95 ± 0.23**	**11.89**
**AP**	**1.73 ± 0.01 ^b^**	**2.01 ± 0.03 ^a^**	**1.50 ± 0.04 ^c^**	**1.75 ± 0.25**	**14.56**	Emcapa 153	1.25 ± 0.01 ^a^	1.31 ± 0.02 ^a^	1.28 ± 0.06 ^a^	1.28 ± 0.03	2.25
**L80**	**1.59 ± 0.02 ^b^**	**1.77 ± 0.02 ^a^**	**1.65 ± 0.04 ^b^**	**1.67 ± 0.09**	**5.62**	P1	0.92 ± 0.03 ^c^	1.09 ± 0.02 ^b^	1.15 ± 0.04 ^a^	1.05 ± 0.12	11.37
Bamburral	1.11 ± 0.01 ^c^	1.34 ± 0.02 ^b^	1.39 ± 0.03 ^b^	1.28 ± 0.15	11.82	LB1	1.36 ± 0.01 ^a^	1.39 ± 0.02 ^a^	1.38 ± 0.04 ^a^	1.38 ± 0.02	1.23
Pirata	1.01 ± 0.01 ^c^	1.11 ± 0.01 ^b^	1.27 ± 0.04 ^a^	1.13 ± 0.13	11.58	**122**	**1.93 ± 0.01 ^b^**	**2.09 ± 0.02 ^a^**	**2.07 ± 0.01 ^a^**	**2.03 ± 0.09**	**4.25**
Peneirão	0.99 ± 0.01 ^c^	1.15 ± 0.03 ^b^	1.32 ± 0.04 ^a^	1.15 ± 0.17	14.42	Verdim D	1.38 ± 0.02 ^b^	1.45 ± 0.02 ^a^	1.44 ± 0.03 ^a^	1.42 ± 0.04	2.87
Z39	1.22 ± 0.03 ^b^	1.37 ± 0.02 ^a^	1.40 ± 0.01 ^a^	1.33 ± 0.09	6.99	Emcapa 143	0.80 ± 0.02 ^c^	0.87 ± 0.02 ^b^	1.56 ± 0.03 ^a^	1.08 ± 0.42	39.15
Z35	0.95 ± 0.02 ^b^	0.97 ± 0.05 ^b^	1.27 ± 0.02 ^a^	1.06 ± 0.18	16.63	Ouro negro 1	1.29 ± 0.02 ^c^	1.39 ± 0.01 ^b^	1.74 ± 0.01 ^a^	1.47 ± 0.24	16.17
Z40	0.96 ± 0.01 ^b^	0.94 ± 0.05 ^b^	1.19 ± 0.02 ^a^	1.03 ± 0.14	13.64	Ouro negro 2	1.03 ± 0.03 ^b^	0.97 ± 0.01 ^c^	1.35 ± 0.02 ^a^	1.12 ± 0.21	18.52
Z29	1.11 ± 0.02 ^b^	1.05 ± 0.07 ^b^	1.29 ± 0.05 ^a^	1.15 ± 0.12	10.52	**Clementino**	**1.77 ± 0.03 ^b^**	**1.84 ± 0.05 ^a^**	**1.74 ± 0.04 ^b^**	**1.78 ± 0.05**	**2.71**
Note (**c**): * Average total CGA contents of the three consecutive crops (2018, 2019, and 2020). Total CGA: sum of CQA + FQA + diCQA. CQA: sum of 3-CQA (3-caffeoylquinic acid) + 4-CQA (4-caffeoylquinic acid) + 5-CQA (5-caffeoylquinic acid). FQA: sum of 4-FQA (4-feruloylquinic acid) + 5-FQA (5-feruloylquinic acid). diCQA: sum of 3,4-diCQA (3,4-dicaffeoylquinic acid) + 3,5-diCQA (3,5-dicaffeoylquinic acid) + 4,5-diCQA (4,5-dicaffeoylquinic acid). CV: coefficient of variation. Different letters for the same genotype indicate statistical differences between crops by ANOVA (*p* < 0.05). Values in bold were consistently outstanding compared to other genotypes.											

#### 2.3.2. Caffeine

The caffeine contents in the green seeds, husks, and leaves from three consecutive crops of conilon plants are presented in Table 4a–c. The contents observed in the seeds (Table 4a) ranged between 1.21 and 2.63 g/100 g db are in accordance with previous reports (1.5–2.5 g/100 g db) for conilon seeds [36]. The contents in husks (Table 4b) ranged between 0.13 and 0.84 g/100 g db, and in leaves (Table 4c), between 0.33 and 2.01 g/100 g. On average, the contents in husks and leaves were 75% and 63% lower (*p* = 0.001), respectively, than those observed in the seeds, indicating concentration of caffeine in the seeds.

Caffeine function in coffee plants and seeds seems to be related to protection. The “chemical defence theory” proposes that caffeine in young leaves, fruits and flower buds act to protect soft tissues from predators such as insect larvae [37] and beetles [38]. The “allelopathic theory” proposes that caffeine in seeds coats is released into the soil and inhibits the germination of other seeds [39].

Chaves et al. [40] evaluated caffeine content in seeds and leaves of arabica and canefora cultivars. They reported average contents in husks of robusta samples (0.25 g/100 g), lower than those observed for the conilon genotypes in this study. Lower contents were also reported for adult robusta leaves from Uganda (0.28–0.75 g/100 g db) [18]. Chaves et al. [40] suggested a mechanism for transporting nitrogen from the leaves to the fruits during their maturation, which is responsible for the high caffeine content in the seeds of these species. The authors observed that the caffeine concentration in the seeds increased linearly about the first and third pairs of leaves. They also reported that the contents in the leaves of arabica and canefora species reduced during the fruit maturation, indicating the redistribution of caffeine and its transport from the leaves to the fruit development. The authors suggested that the first and third pairs of leaves can efficiently and time-savingly predict the caffeine content in the seeds of these same plants when they are adults.

Regarding the variability over the three crops, the CV% ranged from 2.0% to 11.7% in seeds, with the majority (25 genotypes) lower than 5%. The CV variation in husks was 3.6–25.5% and in leaves 2.6–33.6%. The large variation found for a few genotypes was probably caused by edaphoclimatic conditions and the active distribution of caffeine through the different parts of the plant [25].

Still in Table 4a, outstanding mean caffeine contents were observed in seven seeds genotypes (in bold), compared to previous reports in the literature [36], varying between 2.10 and 2.51 g/100 g db, about 25–50% higher than the mean value for the remaining genotypes. Among husks (Table 4b), prominent mean contents were observed in five genotypes, varying between 1.18 and 1.69 g/100 g db, which is 4 to 5 times higher than those observed in some genotypes and higher than those observed in arabica seeds. Among the leaves (Table 4c), eight genotypes stood out, with mean contents between 1.27 and 1.59 g/100 g db, 40% to 80% higher than the contents of the leaves of the remaining genotypes and higher than the contents in arabica seeds, from which caffeine is mostly extracted for use in the manufacture of beverages and the pharmaceutical industry. In general, the genotypes Alecrim, Tardio C, and P2 stood out in relation to mean caffeine contents in seeds, husks, and leaves in the three crops evaluated. These genotypes are of great interest in the caffeine industry given that all parts of the plant can be sources of the substance.

**Table 4:** Caffeine contents (g/100 g) in green seeds (**a**), husks (**b**) and leaves (**c**) of selected *C. canephora* cv. conilon genotypes, from three consecutive crops.

(**a**)											
**Genotype**	***Crop 1***	***Crop 2***	***Crop 3***	Mean *	***CV (%)***	**Genotype**	***Crop 1***	***Crop 2***	***Crop 3***	Mean *	***CV (%)***
Verdim R	2.08 ± 0.02 ^a^	1.95 ± 0.02 ^b^	1.94 ± 0.02 ^b^	1.99 ± 0.08	4.07	Z38	1.81 ± 0.01 ^a^	1.77 ± 0.02 ^b^	1.56 ± 0.02 ^c^	1.71 ± 0.13	7.71
**B01**	**2.32 ± 0.02 ^a^**	**2.13 ± 0.02 ^b^**	**2.15 ± 0.01 ^b^**	**2.20 ± 0.10**	**4.58**	Z18	1.88 ± 0.01 ^a^	1.60 ± 0.02 ^b^	1.56 ± 0.01 ^c^	1.68 ± 0.18	10.47
Bicudo	1.87 ± 0.02 ^a^	1.79 ± 0.01 ^b^	1.84 ± 0.04 ^a^	1.83 ± 0.04	1.99	Z17	1.80 ± 0.03 ^a^	1.84 ± 0.01 ^a^	1.70 ± 0.02 ^b^	1.78 ± 0.07	4.09
**Alecrim**	**2.23 ± 0.02 ^b^**	**2.21 ± 0.01 ^b^**	**2.33 ± 0.04 ^a^**	**2.26 ± 0.07**	**2.99**	Z21	1.86 ± 0.02 ^c^	1.93 ± 0.02 ^b^	2.00 ± 0.02 ^a^	1.93 ± 0.07	3.44
700	1.74 ± 0.05 ^b^	1.90 ± 0.01 ^a^	1.79 ± 0.04 ^b^	1.81 ± 0.08	4.53	Z36	1.82 ± 0.02 ^a^	1.74 ± 0.02 ^b^	1.62 ± 0.02 ^c^	1.73 ± 0.10	5.84
CH1	1.70 ± 0.02 ^b^	1.74 ± 0.01 ^a^	1.54 ± 0.02 ^c^	1.66 ± 0.10	6.29	Ouro negro	1.63 ± 0.03 ^b^	1.54 ± 0.02 ^c^	1.82 ± 0.03 ^a^	1.66 ± 0.15	8.78
**Imbigudinho**	**2.15 ± 0.03 ^b^**	**2.23 ± 0.01 ^a^**	**1.93 ± 0.03 ^c^**	**2.10 ± 0.16**	**7.43**	18	1.66 ± 0.01 ^b^	1.43 ± 0.02 ^c^	1.76 ± 0.03 ^a^	1.61 ± 0.17	10.48
AT	1.50 ± 0.03 ^c^	1.64 ± 0.01 ^b^	1.72 ± 0.03 ^a^	1.62 ± 0.11	6.91	**Tardio C**	**2.63 ± 0.03 ^a^**	**2.55 ± 0.03 ^b^**	**2.21 ± 0.04 ^c^**	**2.46 ± 0.23**	**9.22**
Graudão HP	2.06 ± 0.04 ^a^	2.12 ± 0.02 ^a^	1.94 ± 0.04 ^b^	2.04 ± 0.09	4.38	A1	1.87 ± 0.02 ^a^	1.77 ± 0.02 ^c^	1.82 ± 0.03 ^b^	1.82 ± 0.05	2.87
Valcir P	1.73 ± 0.02 ^c^	1.81 ± 0.01 ^b^	1.92 ± 0.02 ^a^	1.82 ± 0.10	5.37	**Cheique**	**2.22 ± 0.03 ^a^**	**2.16 ± 0.02 ^b^**	**2.09 ± 0.02 ^c^**	**2.15 ± 0.07**	**3.02**
**Beira Rio 8**	**2.53 ± 0.04 ^a^**	**2.45 ± 0.02 ^b^**	**2.55 ± 0.06 ^a^**	**2.51 ± 0.05**	**2.16**	**P2**	**2.29 ± 0.01 ^a^**	**2.17 ± 0.01 ^c^**	**2.24 ± 0.03 ^b^**	**2.23 ± 0.06**	**2.62**
Tardio V	2.25 ± 0.02 ^a^	2.29 ± 0.03 ^a^	1.90 ± 0.02 ^b^	2.15 ± 0.21	9.92	Emcapa 02	1.72 ± 0.03 ^b^	1.53 ± 0.02 ^c^	1.85 ± 0.03 ^a^	1.70 ± 0.16	9.58
AP	2.03 ± 0.01 ^b^	2.13 ± 0.02 ^a^	1.95 ± 0.05 ^c^	2.04 ± 0.09	4.42	Emcapa 153	1.55 ± 0.03 ^b^	1.41 ± 0.02 ^c^	1.71 ± 0.02 ^a^	1.56 ± 0.15	9.63
L80	1.21 ± 0.02 ^c^	1.42 ± 0.02 ^a^	1.35 ± 0.03 ^b^	1.33 ± 0.11	8.02	P1	1.88 ± 0.01 ^a^	1.83 ± 0.02 ^b^	1.80 ± 0.02 ^b^	1.83 ± 0.06	3.11
Bamburral	1.96 ± 0.01 ^c^	2.16 ± 0.01 ^b^	2.23 ± 0.04 ^a^	2.12 ± 0.14	6.73	LB1	1.82 ± 0.03 ^a^	1.53 ± 0.02 ^c^	1.66 ± 0.05 ^b^	1.67 ± 0.15	8.79
Pirata	1.60 ± 0.00 ^a^	1.42 ± 0.01 ^b^	1.37 ± 0.03 ^c^	1.46 ± 0.12	8.47	122	2.00 ± 0.01 ^b^	2.11 ± 0.01 ^a^	1.83 ± 0.03 ^c^	1.98 ± 0.14	7.22
Peneirão	1.86 ± 0.01 ^a^	1.67 ± 0.02 ^c^	1.72 ± 0.03 ^b^	1.75 ± 0.10	5.81	Verdim D	1.97 ± 0.01 ^b^	2.12 ± 0.01 ^a^	2.07 ± 0.06 ^a^	2.05 ± 0.07	3.62
Z39	1.63 ± 0.04 ^a^	1.33 ± 0.01 ^b^	1.35 ± 0.05 ^b^	1.44 ± 0.17	11.70	Emcapa 143	1.91 ± 0.02 ^c^	2.14 ± 0.02 ^b^	2.33 ± 0.02 ^a^	2.13 ± 0.21	9.75
Z35	1.60 ± 0.01 ^a b^	1.54 ± 0.02 ^b^	1.72 ± 0.07 ^a^	1.62 ± 0.09	5.62	Ouro negro 1	1.65 ± 0.02 ^c^	1.77 ± 0.01 ^b^	1.87 ± 0.06 ^a^	1.77 ± 0.11	6.19
Z40	1.95 ± 0.02 ^b^	2.17 ± 0.03 ^a^	2.06 ± 0.04 ^c^	2.06 ± 0.11	5.37	Ouro negro 2	1.54 ± 0.01 ^b^	1.44 ± 0.02 ^c^	1.66 ± 0.02 ^a^	1.54 ± 0.11	7.12
Z29	1.81 ± 0.02 ^b^	2.02 ± 0.02 ^a^	1.72 ± 0.03 ^c^	1.85 ± 0.16	8.42	Clementino	1.77 ± 0.01 ^b^	1.77 ± 0.01 ^b^	1.95 ± 0.03 ^a^	1.83 ± 0.10	5.53
Note (**a**): * Average caffeine contents of the three consecutive crops (2018, 2019, and 2020). CV: coefficient of variation. Different letters for the same genotype indicate statistical differences between crops by ANOVA (*p* < 0.05). Values in bold indicate genotypes that stood out for their consistently high contents of caffeine (>2%, CV < 10%) in all crops evaluated, compared to average contents described in the literature [36] and with the remaining genotypes.											
(**b**)											
**Genotype**	***Crop 1***	***Crop 2***	***Crop 3***	Mean *	***CV (%)***	**Genotype**	***Crop 1***	***Crop 2***	***Crop 3***	Mean *	***CV (%)***
Verdim R	1.04 ± 0.03 ^a^	0.94 ± 0.02 ^b^	0.84 ± 0.02 ^c^	0.94 ± 0.10	10.48	Z38	1.15 ± 0.01 ^a^	0.97 ± 0.02 ^b^	0.96 ± 0.01 ^b^	1.03 ± 0.11	10.60
B01	0.85 ± 0.01 ^b^	0.95 ± 0.02 ^a^	0.86 ± 0.01 ^b^	0.88 ± 0.05	6.19	Z18	1.02 ± 0.01 ^a^	0.95 ± 0.03 ^b^	0.81 ± 0.01 ^c^	0.93 ± 0.11	11.46
Bicudo	0.51 ± 0.02 ^b^	0.65 ± 0.04 ^a^	0.63 ± 0.01 ^a^	0.60 ± 0.07	12.29	Z17	0.21 ± 0.02 ^c^	0.37 ± 0.02 ^a^	0.30 ± 0.02 ^b^	0.29 ± 0.08	27.00
**Alecrim**	**1.41 ± 0.01 ^b^**	**1.46 ± 0.02 ^a^**	**1.34 ± 0.02 ^c^**	**1.41 ± 0.06**	**4.28**	Z21	0.22 ± 0.01 ^c^	0.36 ± 0.04 ^a^	0.35 ± 0.01 ^b^	0.31 ± 0.08	24.87
700	0.87 ± 0.01 ^c^	0.95 ± 0.03 ^b^	1.11 ± 0.01 ^a^	0.98 ± 0.12	12.55	Z36	0.45 ± 0.00 ^a^	0.36 ± 0.01 ^b^	0.36 ± 0.02 ^b^	0.39 ± 0.05	13.66
CH1	0.38 ± 0.04 ^c^	0.46 ± 0.03 ^a^	0.44 ± 0.03 ^b^	0.43 ± 0.04	9.14	Ouro negro	0.88 ± 0.00 ^c^	1.02 ± 0.01 ^a^	0.95 ± 0.05 ^b^	0.95 ± 0.07	7.52
**Imbigudinho**	**1.22 ± 0.02 ^b^**	**1.14 ± 0.01 ^c^**	**1.32 ± 0.02 ^a^**	**1.23 ± 0.09**	**7.22**	18	0.90 ± 0.01 ^b^	0.95 ± 0.02 ^a^	0.86 ± 0.03 ^c^	0.91 ± 0.05	4.98
AT	0.81 ± 0.02 ^b^	0.92 ± 0.02 ^a^	0.84 ± 0.02 ^b^	0.86 ± 0.06	6.85	**Tardio C**	**1.70 ± 0.01 ^b^**	**1.84 ± 0.02 ^a^**	**1.53 ± 0.02 ^c^**	**1.69 ± 0.16**	**9.21**
Graudão HP	0.92 ± 0.01 ^a^	0.73 ± 0.02 ^b^	0.75 ± 0.01 ^b^	0.80 ± 0.10	13.02	A1	0.22 ± 0.01 ^a^	0.24 ± 0.01 ^a^	0.15 ± 0.03 ^b^	0.21 ± 0.05	22.45
Valcir P	0.22 ± 0.02 ^b^	0.28 ± 0.01 ^a^	0.30 ± 0.02 ^a^	0.27 ± 0.04	14.91	Cheique	0.44 ± 0.01 ^a^	0.47 ± 0.01 ^a^	0.25 ± 0.02 ^b^	0.39 ± 0.12	30.98
Beira Rio 8	0.87 ± 0.02 ^a^	0.88 ± 0.02 ^a^	0.76 ± 0.02 ^b^	0.83 ± 0.07	7.79	**P2**	**1.23 ± 0.01 ^a^**	**1.16 ± 0.01 ^b^**	**1.16 ± 0.02 ^b^**	**1.18 ± 0.04**	**3.61**
Tardio V	1.10 ± 0.02 ^b^	1.04 ± 0.01 ^c^	1.15 ± 0.03 ^a^	1.09 ± 0.05	4.88	Emcapa 02	0.70 ± 0.01 ^b^	0.85 ± 0.01 ^a^	0.83 ± 0.02 ^a^	0.79 ± 0.08	10.66
AP	0.89 ± 0.01 ^b^	0.95 ± 0.02 ^a^	0.77 ± 0.01 ^c^	0.87 ± 0.09	10.54	Emcapa 153	1.07 ± 0.01 ^b^	1.16 ± 0.02 ^a^	0.84 ± 0.02 ^c^	1.03 ± 0.17	16.29
L80	0.55 ± 0.02 ^b^	0.65 ± 0.02 ^a^	0.55 ± 0.02 ^b^	0.59 ± 0.06	9.85	P1	0.63 ± 0.00 ^b^	0.74 ± 0.02 ^a^	0.72 ± 0.02 ^a^	0.70 ± 0.06	8.01
**Bamburral**	**1.35 ± 0.01 ^b^**	**1.24 ± 0.03 ^c^**	**1.44 ± 0.02 ^a^**	**1.34 ± 0.10**	**7.21**	LB1	1.00 ± 0.01 ^a^	1.02 ± 0.02 ^a^	0.64 ± 0.02 ^b^	0.89 ± 0.21	24.09
Pirata	0.31 ± 0.03 ^b^	0.42 ± 0.02 ^a^	0.33 ± 0.03 ^b^	0.35 ± 0.06	16.23	122	1.03 ± 0.01 ^b^	1.10 ± 0.02 ^a^	0.78 ± 0.02 ^c^	0.97 ± 0.17	17.20
Peneirão	1.11 ± 0.01 ^b^	1.02 ± 0.02 ^c^	1.25 ± 0.04 ^a^	1.13 ± 0.11	10.13	Verdim D	0.87 ± 0.02 ^a^	0.99 ± 0.01 ^b^	1.04 ± 0.03 ^b^	0.96 ± 0.08	8.78
Z39	0.40 ± 0.02 ^a^	0.41 ± 0.03 ^a^	0.45 ± 0.04 ^a^	0.42 ± 0.03	6.46	Emcapa 143	0.62 ± 0.02 ^b^	0.86 ± 0.01 ^a^	0.62 ± 0.03 ^b^	0.70 ± 0.14	19.80
Z35	0.13 ± 0.00 ^c^	0.20 ± 0.01 ^a^	0.17 ± 0.01 ^b^	0.17 ± 0.03	20.08	Ouro negro 1	1.08 ± 0.01 ^a^	1.11 ± 0.01 ^a^	0.82 ± 0.02 ^b^	1.00 ± 0.16	15.69
Z40	0.22 ± 0.01 ^b^	0.24 ± 0.03 ^b^	0.35 ± 0.01 ^a^	0.27 ± 0.07	25.54	Ouro negro 2	1.12 ± 0.01 ^a^	1.10 ± 0.01 ^b^	0.83 ± 0.01 ^c^	1.01 ± 0.16	15.78
Z29	1.13 ± 0.01 ^a^	1.03 ± 0.01 ^b^	0.82 ± 0.02 ^c^	0.99 ± 0.16	16.08	Clementino	0.41 ± 0.01 ^c^	0.57 ± 0.02 ^a^	0.47 ± 0.02 ^b^	0.48 ± 0.08	17.14
Note (**b**): * Average caffeine contents of the three consecutive crops (2018, 2019, and 2020). CV: coefficient of variation. Different letters for the same genotype indicate statistical differences between crops by ANOVA (*p* < 0.05). Values in bold were consistently outstanding compared to other genotypes.											
(**c**)											
**Genotype**	***Crop 1***	***Crop 2***	***Crop 3***	Mean *	***CV (%)***	**Genotype**	***Crop***	***Crop 2***	***Crop 3***	Mean *	***CV (%)***
Verdim R	1.50 ± 0.01 ^a^	1.12 ± 0.02 ^b^	1.07 ± 0.02 ^c^	1.23 ± 0.24	19.34	Z38	1.17 ± 0.00 ^a^	1.18 ± 0.02 ^a^	0.97 ± 0.02 ^b^	1.11 ± 0.12	10.50
B01	1.41 ± 0.02 ^a^	1.15 ± 0.02 ^b^	0.96 ± 0.03 ^c^	1.17 ± 0.23	19.22	**Z18**	**1.59 ± 0.01 ^a^**	**1.61 ± 0.01 ^a^**	**1.26 ± 0.03 ^b^**	**1.49 ± 0.20**	**13.29**
Bicudo	0.87 ± 0.01 ^b^	0.93 ± 0.01 ^a^	0.95 ± 0.03 ^a^	0.92 ± 0.04	4.40	Z17	0.41 ± 0.01 ^c^	0.63 ± 0.02 ^a^	0.55 ± 0.03 ^b^	0.53 ± 0.11	21.28
**Alecrim**	**1.44 ± 0.01 ^a^**	**1.25 ± 0.01 ^b^**	**1.44 ± 0.02 ^a^**	**1.38 ± 0.11**	**8.13**	Z21	1.07 ± 0.02 ^b^	1.13 ± 0.03 ^a^	1.11 ± 0.02 ^a^	1.11 ± 0.03	2.58
700	1.01 ± 0.01 ^a^	1.04 ± 0.02 ^a^	0.96 ± 0.02 ^b^	1.01 ± 0.04	3.99	Z36	1.20 ± 0.01 ^b^	1.22 ± 0.01 ^b^	1.26 ± 0.02 ^a^	1.23 ± 0.03	2.78
CH1	0.85 ± 0.01 ^c^	0.96 ± 0.02 ^b^	1.06 ± 0.01 ^a^	0.96 ± 0.11	11.15	Ouro negro	1.37 ± 0.01 ^a^	1.20 ± 0.02 ^b^	1.02 ± 0.03 ^c^	1.20 ± 0.17	14.61
**Imbigudinho**	**1.64 ± 0.03 ^b^**	**1.78 ± 0.02 ^a^**	**1.26 ± 0.02 ^c^**	**1.56 ± 0.27**	**17.23**	18	1.44 ± 0.01 ^a^	1.06 ± 0.01 ^b^	1.04 ± 0.02 ^b^	1.18 ± 0.22	19.02
AT	0.94 ± 0.01 ^b^	1.00 ± 0.05 ^a b^	1.07 ± 0.03 ^a^	1.01 ± 0.06	6.24	**Tardio C**	**1.42 ± 0.01 ^a^**	**1.21 ± 0.03 ^b^**	**1.16 ± 0.03 ^b^**	**1.27 ± 0.14**	**10.88**
Graudão HP	1.42 ± 0.02 ^a^	1.34 ± 0.02 ^b^	1.15 ± 0.01 ^c^	1.30 ± 0.14	10.53	A1	1.03 ± 0.01 ^c^	1.14 ± 0.02 ^b^	1.28 ± 0.03 ^a^	1.15 ± 0.13	10.97
Valcir P	0.65 ± 0.01 ^b^	0.76 ± 0.02 ^a^	0.56 ± 0.00 ^c^	0.66 ± 0.10	15.46	Cheique	1.07 ± 0.01 ^b^	1.14 ± 0.02 ^a^	1.10 ± 0.04 ^b^	1.10 ± 0.03	3.00
Beira Rio 8	1.01 ± 0.01 ^a^	1.03 ± 0.01 ^a^	0.94 ± 0.02 ^b^	0.99 ± 0.05	4.73	**P2**	**1.40 ± 0.01 ^c^**	**1.51 ± 0.02 ^b^**	**1.85 ± 0.03 ^a^**	**1.59 ± 0.23**	**14.73**
Tardio V	0.33 ± 0.01 ^c^	0.44 ± 0.02 ^b^	0.54 ± 0.02 ^a^	0.44 ± 0.11	24.65	Emcapa 02	1.76 ± 0.01 ^b^	1.82 ± 0.01 ^a^	1.02 ± 0.04 ^c^	1.53 ± 0.45	29.27
**AP**	**2.01 ± 0.02 ^a^**	**1.78 ± 0.02 ^b^**	**1.81 ± 0.03 ^c^**	**1.87 ± 0.13**	**6.78**	Emcapa 153	1.10 ± 0.01 ^c^	1.50 ± 0.02 ^b^	1.66 ± 0.04 ^a^	1.42 ± 0.29	20.20
L80	0.86 ± 0.02 ^b^	0.93 ± 0.03 ^a^	0.74 ± 0.01 ^c^	0.84 ± 0.09	11.23	P1	0.81 ± 0.01 ^c^	0.94 ± 0.02 ^b^	0.99 ± 0.03 ^a^	0.91 ± 0.09	10.11
**Bamburral**	**1.47 ± 0.02 ^b^**	**1.56 ± 0.03 ^a^**	**1.36 ± 0.02 ^c^**	**1.46 ± 0.10**	**7.07**	LB1	0.96 ± 0.01 ^b^	1.04 ± 0.02 ^a^	0.86 ± 0.04 ^c^	0.95 ± 0.09	9.65
Pirata	0.59 ± 0.02 ^c^	0.77 ± 0.02 ^b^	0.86 ± 0.03 ^a^	0.74 ± 0.14	18.28	122	1.12 ± 0.02 ^b^	1.13 ± 0.03 ^b^	1.34 ± 0.02 ^a^	1.20 ± 0.13	10.52
Peneirão	0.82 ± 0.02 ^b^	0.92 ± 0.02 ^a^	0.77 ± 0.02 ^c^	0.83 ± 0.07	8.68	Verdim D	1.29 ± 0.03 ^a^	1.10 ± 0.01 ^b^	0.83 ± 0.02 ^c^	1.07 ± 0.23	21.38
Z39	0.79 ± 0.01 ^b^	1.02 ± 0.02 ^a^	1.05 ± 0.03 ^a^	0.95 ± 0.14	14.96	Emcapa 143	1.11 ± 0.02 ^b^	1.16 ± 0.02 ^a^	0.97 ± 0.01 ^c^	1.08 ± 0.10	9.24
Z35	0.35 ± 0.02 ^c^	0.67 ± 0.02 ^a^	0.44 ± 0.04 ^b^	0.49 ± 0.16	33.55	Ouro negro 1	1.00 ± 0.01 ^a^	1.06 ± 0.03 ^a^	0.86 ± 0.03 ^b^	0.97 ± 0.10	10.57
Z40	0.79 ± 0.01 ^a b^	0.90 ± 0.07 ^a^	0.76 ± 0.03 ^b^	0.82 ± 0.07	9.10	Ouro negro 2	1.66 ± 0.03 ^b^	1.72 ± 0.02 ^a^	1.07 ± 0.03 ^c^	1.48 ± 0.36	24.36
**Z29**	**1.50 ± 0.01 ^b^**	**1.69 ± 0.01 ^a^**	**1.24 ± 0.03 ^c^**	**1.47 ± 0.23**	**15.33**	Clementino	0.57 ± 0.02 ^c^	0.77 ± 0.02 ^b^	0.87 ± 0.02 ^a^	0.74 ± 0.15	20.64
Note (**c**): * Average caffeine contents of the three consecutive crops (2018, 2019, and 2020). CV: coefficient of variation. Different letters for the same genotype indicate statistical differences between crops by ANOVA (*p* < 0.05). Values in bold were consistently outstanding compared to other genotypes.											

#### 2.3.3. Trigonelline

The mean trigonelline contents in the seeds, husks, and leaves from three consecutive crops of conilon genotypes are presented in Table 5a–c. In general, differently from CGA and caffeine, considering the dry basis, trigonelline was shown to be similarly distributed in seeds, husks, and leaves.

In Table 5a, the contents in seeds varied from 0.83 to 1.12 g/100 g db. These contents are similar to those found in previous reports for conilon coffee seeds (0.52–1.24 g/100 g db) [41]. Although canefora coffees are not typically good sources of trigonelline compared to arabica coffees, outstanding mean contents in seeds were observed in thirteen genotypes (in bold) compared to those described in the literature [41], varying between 1.03 and 1.09 g/100 g db, about 25–30% higher than the mean value for the remaining genotypes. These outstanding contents are comparable to those found in arabica plants [41].

The contents in husks (Table 5b) ranged between 0.59 and 1.24 g/100 g db, and in leaves (Table 5c) between 0.74 and 1.84 g/100 g db. Lower values have been reported for robusta leaves from Uganda (0.42–0.60 g/100 g db) [18]. No report on trigonelline content in conilon husks was found for comparison. Among husks, prominent mean contents were observed in seven genotypes and varied between 1.05 and 1.15 g/100 g db, 2 to 2.5 times higher than those observed in some of the remaining genotypes. Regarding the leaves, fourteen genotypes stood out, with mean contents varying between 1.30 and 1.77 g/100 g db, higher or similar to the contents commonly found in arabica seeds (0.80–1.20 g/100 g db), which are usually higher than in canefora seeds [41]. In general, the genotypes Valcir P and Cheique stood out about trigonelline contents in seeds, husks, and leaves in the three crops evaluated. These genotypes are of great interest by industry given that all parts of the plant can be sources of the substance.

Regarding the variability among the three crops, the CV% ranged from 0.48% to 9.95% in seeds, 1.49% to 22.16% in husks, and 1.15% to 19.41% in leaves. As with the other parts of the plant, this variation was probably caused by the edaphoclimatic and physiological conditions [31]. Several plant roles for trigonelline have been proposed: as a nutrient source, a compatible solute, a bioactive substance for nyctinasty, cell cycle regulation, signal transduction, detoxification of nicotinic acid, and for eco-chemical functions such as host selection by herbivores [42].

**Figure 1:**
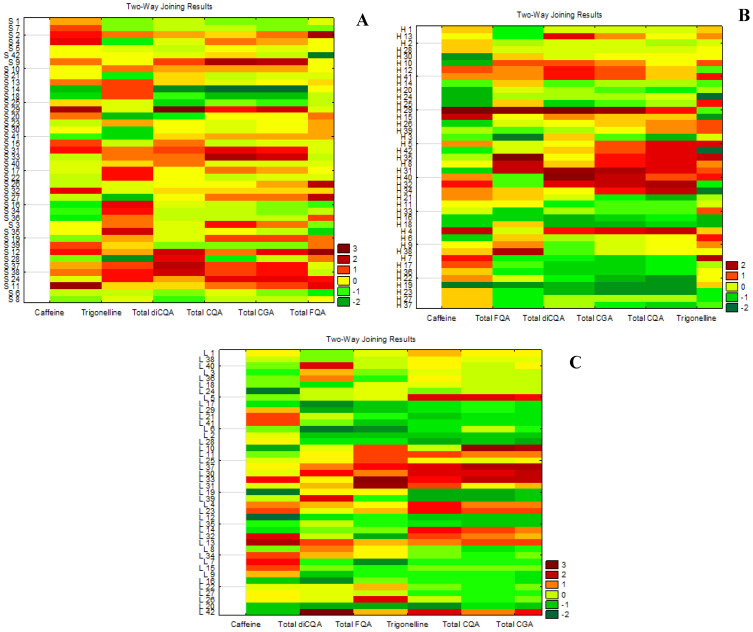
Heat maps built using multivariate analysis based on the contents of bioactive compounds in the seeds, husks and leaves of the studied conilon genotypes, considering all. CGA: total chlorogenic acids; diCQA: dicafeoylquinic acids; FQA: feruloylquinic acids; (**A**): S (seeds); (**B**): H (husks), (**C**): L (leaves). Names of the genotypes—1: Verdim R; 2: B01; 3: Bicudo; 4: Alecrim; 5: 700; 6: CH1; 7: Imbigudinho; 8: AT; 9: Graudão HP; 10: Valcir P; 11: Beira Rio 8; 12: Tardio V; 13: AP; 14: L80; 15: Bamburral; 16: Pirata; 17: Peneirão; 18: Z39; 19: Z35; 20: Z40; 21: Z29; 22: Z38; 23: Z18; 24: Z17; 25: Z21; 26: Z36; 27: Ouro negro; 28: 18; 29: Tardio C; 30: A1; 31: Cheique; 32: P2; 33: Emcapa 02; 34: Emcapa 153; 35: P1; 36: LB1; 3: 7: 122; 38: Verdim D; 39: Emcapa 143; 40: Ouro negro 1; 41: Ouro negro 2; 42: Clementino.

Figure 1 shows heat maps built using multivariate analysis based on the bioactive compound contents in the seeds, husks, and leaves of the studied genotypes, summarizing the contents presented in Table 3, Table 4 and Table 5. The values in dark brown indicate the highest average contents of the bioactive compound considering all crops. Dark green represents the lowest contents. The CGA are presented as total content (total CGA) and by classes (total CQA, total FQA, and total diCQA). It is worth noting that some genotypes presented higher amounts of CQA, diCQA, and or FQA. These classes have been identified in several medicinal plants, especially the diCQA, which are characteristic of propolis extract of the Atlantic rain forest [43,44], *Ilex kudingcha* [45], *Hedera helix*, *Echinacea sp* and have immune-stimulant, anti-inflammatory and antioxidant properties, among others [46,47]. CQA, FQA, and diCQA are also abundant in *Ilex paraguariensis* (yerba mate) plants, which are widely consumed in South America. FQA have not been as studied as the isomers of other CGA classes, but they are potent antioxidants [48] and have been identified in several medicinal plants, for example, *Baccharis genisteloides*, *Pimpinella anisum* and *Cymbopogon citratus* [49]. Therefore, in the future, some of these genotypes may have a special interest for medicinal purposes.

**Table 5:** Trigonelline contents (g/100 g) in green seeds seeds (**a**), husks (**b**) and leaves (**c**) of selected *C. canephora* cv. conilon genotypes, from three consecutive crops.

(**a**)											
**Genotype**	***Crop 1***	***Crop 2***	***Crop 3***	Mean *	***CV (%)***	**Genotype**	***Crop 1***	***Crop 2***	***Crop 3***	Mean *	***CV (%)***
Verdim R	0.93 ± 0.03 ^a^	0.94 ± 0.01 ^a^	0.89 ± 0.02 ^b^	0.92 ± 0.03	2.89	**Z38**	**1.05 ± 0.02 ^a^**	**1.03 ± 0.03 ^b^**	**1.06 ± 0.01 ^a^**	**1.05 ± 0.02**	**1.63**
B01	0.95 ± 0.02 ^c^	1.04 ± 0.02 ^b^	1.08 ± 0.02 ^a^	1.02 ± 0.07	6.56	Z18	1.10 ± 0.02 ^a^	0.95 ± 0.02 ^b c^	0.99 ± 0.03 ^b^	1.01 ± 0.08	7.98
Bicudo	0.96 ± 0.03 ^c^	1.03 ± 0.02 ^b^	1.07 ± 0.02 ^a^	1.02 ± 0.05	5.29	**Z17**	**1.06 ± 0.02 ^a^**	**1.01 ± 0.01 ^b^**	**1.07 ± 0.01 ^a^**	**1.05 ± 0.03**	**3.06**
Alecrim	0.88 ± 0.02 ^b^	0.95 ± 0.02 ^a^	0.83 ± 0.02 ^b c^	0.89 ± 0.06	6.77	Z21	1.02 ± 0.01 ^a^	0.94 ± 0.02 ^b^	0.90 ± 0.03 ^c^	0.95 ± 0.06	6.71
700	0.97 ± 0.02 ^b^	1.01 ± 0.01 ^a^	0.91 ± 0.00 ^c^	0.96 ± 0.05	5.10	Z36	1.02 ± 0.02 ^a^	0.96 ± 0.02 ^b^	0.88 ± 0.03 ^c^	0.95 ± 0.07	7.53
CH1	0.91 ± 0.02 ^b^	0.96 ± 0.01 ^a^	0.93 ± 0.04 ^a b^	0.93 ± 0.03	2.70	Ouro negro	0.87 ± 0.01 ^a^	0.81 ± 0.01 ^b^	0.87 ± 0.02 ^a^	0.85 ± 0.03	3.97
Imbigudinho	0.94 ± 0.02 ^a^	0.94 ± 0.02 ^a^	0.88 ± 0.02 ^b^	0.92 ± 0.03	3.67	18	0.82 ± 0.03 ^b^	0.87 ± 0.04 ^a^	0.78 ± 0.03 ^c^	0.82 ± 0.05	5.86
AT	0.95 ± 0.04 ^b^	0.86 ± 0.01 ^c^	1.05 ± 0.03 ^a^	0.96 ± 0.10	9.95	Tardio C	0.96 ± 0.03 ^b^	0.89 ± 0.03 ^c^	1.01 ± 0.04 ^a^	0.95 ± 0.06	6.32
Graudão HP	1.03 ± 0.01 ^a^	0.87 ± 0.03 ^b^	1.01 ± 0.01 ^a^	0.97 ± 0.09	9.10	A1	0.85 ± 0.02 ^b^	0.88 ± 0.01 ^a^	0.91 ± 0.01 ^a^	0.88 ± 0.03	3.60
**Valcir P**	**1.03 ± 0.04 ^a^**	**1.02 ± 0.01 ^a^**	**1.03 ± 0.01 ^a^**	**1.03 ± 0.01**	**0.75**	**Cheique**	**1.05 ± 0.01 ^a^**	**1.00 ± 0.02 ^b^**	**1.03 ± 0.02 ^a b^**	**1.03 ± 0.03**	**2.48**
Beira Rio 8	1.03 ± 0.02 ^a^	0.93 ± 0.01 ^c^	1.00 ± 0.03 ^a b^	0.99 ± 0.05	5.17	P2	1.03 ± 0.02 ^a^	0.98 ± 0.03 ^b^	0.86 ± 0.03 ^c^	0.96 ± 0.09	8.97
Tardio V	1.05 ± 0.01 ^a^	0.95 ± 0.02 ^c^	1.01 ± 0.02 ^b^	1.00 ± 0.05	5.36	**Emcapa 02**	**1.04 ± 0.02 ^a^**	**1.01 ± 0.02 ^b^**	**1.04 ± 0.01 ^a^**	**1.03 ± 0.02**	**1.97**
**AP**	**1.04 ± 0.02 ^a b^**	**1.01 ± 0.02 ^b^**	**1.07 ± 0.02 ^a^**	**1.04 ± 0.03**	**2.74**	**Emcapa 153**	**1.06 ± 0.01 ^a^**	**1.04 ± 0.02 ^b^**	**1.07 ± 0.02 ^a^**	**1.06 ± 0.01**	**1.14**
L80	1.06 ± 0.02 ^a^	0.97 ± 0.03 ^b^	1.07 ± 0.01 ^a^	1.03 ± 0.06	5.45	**P1**	**1.11 ± 0.02 ^a^**	**1.06 ± 0.02 ^b^**	**1.12 ± 0.02 ^a^**	**1.09 ± 0.03**	**2.94**
Bamburral	0.86 ± 0.04 ^c^	0.93 ± 0.04 ^b^	1.04 ± 0.01 ^a^	0.94 ± 0.09	9.41	LB1	1.02 ± 0.01 ^b^	1.06 ± 0.03 ^a^	0.99 ± 0.02 ^b^	1.03 ± 0.04	3.42
**Pirata**	**1.06 ± 0.04 ^a^**	**1.06 ± 0.03 ^a^**	**1.07 ± 0.02 ^a^**	**1.06 ± 0.01**	**0.79**	**122**	**1.03 ± 0.02 ^b^**	**1.02 ± 0.03 ^b^**	**1.07 ± 0.03 ^a^**	**1.04 ± 0.03**	**2.69**
**Peneirão**	**1.06 ± 0.02 ^a^**	**1.06 ± 0.03 ^a^**	**1.05 ± 0.02 ^a^**	**1.06 ± 0.01**	**0.48**	**Verdim D**	**1.05 ± 0.02 ^a^**	**1.05 ± 0.03 ^a^**	**1.05 ± 0.03 ^a^**	**1.05 ± 0.00**	**0.32**
**Z39**	**1.04 ± 0.02 ^a^**	**1.04 ± 0.04 ^a^**	**1.03 ± 0.03 ^a^**	**1.04 ± 0.01**	**0.56**	Emcapa 143	1.06 ± 0.02 ^a^	0.95 ± 0.02 ^b^	0.98 ± 0.02 ^b^	0.99 ± 0.06	5.58
Z35	1.09 ± 0.03 ^a^	0.96 ± 0.03 ^b^	0.96 ± 0.04 ^b^	1.00 ± 0.08	7.48	Ouro negro 1	1.03 ± 0.02 ^a^	0.98 ± 0.05 ^b^	0.93 ± 0.03 ^c^	0.98 ± 0.05	5.09
Z40	0.85 ± 0.01 ^a b^	0.85 ± 0.03 ^a b^	0.88 ± 0.02 ^a^	0.86 ± 0.02	2.14	Ouro negro 2	0.95 ± 0.04 ^a^	0.81 ± 0.02 ^c^	0.86 ± 0.02 ^b^	0.87 ± 0.07	8.35
Z29	0.94 ± 0.02 ^a^	0.83 ± 0.02 ^b^	0.92 ± 0.02 ^a^	0.90 ± 0.05	6.12	Clementino	1.03 ± 0.02 ^a^	0.93 ± 0.02 ^b^	0.95 ± 0.03 ^b^	0.97 ± 0.05	5.32
Note (**a**): * Average trigonelline contents of the three consecutive crops (2018, 2019, and 2020). CV: coefficient of variation. Different letters for the same genotype indicate statistical differences between crops by ANOVA (*p* < 0.05). Values in bold indicate genotypes that stood out for their consistently high contents of trigonelline (>1.0%, CV < 5%) in all crops evaluated, compared to average contents described in the literature [41] and with the remaining genotypes.											
(**b**)											
**Genotype**	***Crop 1***	***Crop 2***	***Crop 3***	Mean *	***CV (%)***	**Genotype**	***Crop 1***	***Crop 2***	***Crop 3***	Mean *	***CV (%)***
Verdim R	0.90 ± 0.02 ^b^	1.04 ± 0.01 ^a^	1.06 ± 0.03 ^a^	1.00 ± 0.08	8.34	Z38	1.03 ± 0.03 ^a^	0.95 ± 0.02 ^b^	0.86 ± 0.01 ^c^	0.95 ± 0.09	9.02
B01	0.96 ± 0.02 ^a^	0.86 ± 0.01 ^b^	0.97 ± 0.05 ^a^	0.93 ± 0.06	6.50	Z18	0.76 ± 0.04 ^c^	0.95 ± 0.02 ^a^	0.82 ± 0.02 ^b^	0.84 ± 0.10	11.46
Bicudo	0.92 ± 0.01 ^b^	0.95 ± 0.01 ^a^	0.93 ± 0.04 ^a b^	0.93 ± 0.01	1.49	Z17	0.59 ± 0.02 ^c^	0.76 ± 0.03 ^a^	0.66 ± 0.01 ^b^	0.67 ± 0.08	12.26
Alecrim	0.96 ± 0.03 ^b^	1.05 ± 0.01 ^a^	0.92 ± 0.02 ^c^	0.98 ± 0.06	6.44	**Z21**	**1.12 ± 0.02 ^a^**	**1.08 ± 0.02 ^b^**	**1.01 ± 0.02 ^c^**	**1.07 ± 0.05**	**4.90**
**700**	**1.04 ± 0.01 ^c^**	**1.13 ± 0.02 ^b^**	**1.19 ± 0.02 ^a^**	**1.12 ± 0.08**	**6.76**	Z36	1.12 ± 0.02 ^a^	1.08 ± 0.02 ^b^	0.95 ± 0.03 ^c^	1.05 ± 0.09	8.49
CH1	1.24 ± 0.02 ^a^	1.08 ± 0.02 ^b^	0.91 ± 0.02 ^c^	1.08 ± 0.16	15.02	Ouro negro	0.87 ± 0.02 ^b^	0.76 ± 0.03 ^b^	0.86 ± 0.04 ^a^	0.83 ± 0.06	7.13
**Imbigudinho**	**1.21 ± 0.02 ^a^**	**1.16 ± 0.02 ^b^**	**1.09 ± 0.02 ^c^**	**1.15 ± 0.06**	**5.53**	18	0.99 ± 0.03 ^a^	0.96 ± 0.02 ^a^	0.88 ± 0.02 ^b^	0.94 ± 0.06	6.10
AT	1.13 ± 0.01 ^a^	1.07 ± 0.02 ^b^	0.87 ± 0.03 ^c^	1.02 ± 0.14	13.50	Tardio C	0.74 ± 0.02 ^c^	0.84 ± 0.02 ^b^	1.00 ± 0.02 ^a^	0.86 ± 0.13	15.20
Graudão HP	1.05 ± 0.03 ^a^	1.05 ± 0.03 ^a^	0.94 ± 0.02 ^b^	1.01 ± 0.06	6.27	A1	0.89 ± 0.02 ^b^	0.89 ± 0.02 ^b^	1.11 ± 0.01 ^a^	0.97 ± 0.13	12.96
**Valcir P**	**1.04 ± 0.02 ^b^**	**1.03 ± 0.02 ^b^**	**1.11 ± 0.02 ^a^**	**1.06 ± 0.05**	**4.48**	**Cheique**	**1.02 ± 0.01 ^b^**	**1.06 ± 0.02 ^a^**	**1.06 ± 0.03 ^a^**	**1.05 ± 0.02**	**2.12**
Beira Rio 8	1.02 ± 0.02 ^a^	0.96 ± 0.03 ^b^	0.83 ± 0.02 ^c^	0.94 ± 0.10	10.69	P2	0.76 ± 0.03 ^c^	0.81 ± 0.01 ^b^	0.97 ± 0.02 ^a^	0.85 ± 0.11	13.13
Tardio V	0.73 ± 0.02 ^c^	0.82 ± 0.03 ^b^	1.01 ± 0.02 ^a^	0.85 ± 0.14	16.56	Emcapa 02	0.96 ± 0.02 ^c^	1.05 ± 0.02 ^b^	1.09 ± 0.04 ^a^	1.03 ± 0.06	6.17
AP	1.02 ± 0.01 ^a^	1.04 ± 0.03 ^a^	0.95 ± 0.03 ^b^	1.00 ± 0.05	4.52	Emcapa 153	0.68 ± 0.02 ^b^	0.72 ± 0.03 ^a^	0.76 ± 0.02 ^a^	0.72 ± 0.04	5.33
L80	0.84 ± 0.03 ^b^	0.92 ± 0.02 ^a^	0.83 ± 0.02 ^b^	0.87 ± 0.05	5.83	**P1**	**1.08 ± 0.02 ^b^**	**1.11 ± 0.02 ^b^**	**1.18 ± 0.02 ^a^**	**1.13 ± 0.05**	**4.60**
Bamburral	0.65 ± 0.02 ^c^	0.72 ± 0.02 ^b^	0.82 ± 0.01 ^a^	0.73 ± 0.08	11.48	LB1	0.86 ± 0.02 ^c^	0.91 ± 0.01 ^b^	1.12 ± 0.02 ^a^	0.96 ± 0.14	14.37
Pirata	0.70 ± 0.02 ^c^	0.84 ± 0.02 ^a^	0.76 ± 0.02 ^b^	0.77 ± 0.07	9.20	122	0.92 ± 0.02 ^a^	0.78 ± 0.02 ^c^	0.87 ± 0.02 ^b^	0.86 ± 0.07	8.64
Peneirão	0.82 ± 0.02 ^c^	0.90 ± 0.03 ^b^	0.93 ± 0.02 ^a^	0.88 ± 0.06	6.33	Verdim D	0.86 ± 0.03 ^c^	0.93 ± 0.03 ^b^	1.07 ± 0.02 ^a^	0.95 ± 0.11	11.26
Z39	0.72 ± 0.02 ^b^	0.62 ± 0.01 ^c^	0.94 ± 0.01 ^a^	0.76 ± 0.16	21.32	Emcapa 143	0.90 ± 0.03 ^c^	1.13 ± 0.03 ^a^	1.08 ± 0.04 ^b^	1.03 ± 0.12	11.75
Z35	0.95 ± 0.03 ^b^	1.04 ± 0.01 ^a^	0.94 ± 0.02 ^b^	0.98 ± 0.06	5.83	Ouro negro 1	0.98 ± 0.02 ^b^	1.10 ± 0.02 ^a^	1.12 ± 0.02 ^a^	1.07 ± 0.08	7.16
Z40	0.67 ± 0.02 ^c^	0.94 ± 0.01 ^b^	1.05 ± 0.02 ^a^	0.88 ± 0.20	22.16	**Ouro negro 2**	**1.04 ± 0.02 ^b^**	**1.13 ± 0.03 ^a^**	**1.05 ± 0.03 ^b^**	**1.08 ± 0.05**	**4.58**
Z29	0.73 ± 0.02 ^c^	0.94 ± 0.02 ^b^	1.02 ± 0.02 ^a^	0.89 ± 0.15	16.90	Clementino	0.64 ± 0.02 ^b^	0.74 ± 0.04 ^a^	0.65 ± 0.03 ^b^	0.68 ± 0.05	7.93
Note (**b**): * Average trigonelline contents of the three consecutive crops (2018, 2019, and 2020). CV: coefficient of variation. Different letters for the same genotype indicate statistical differences between crops by ANOVA (*p* < 0.05). Values in bold were consistently outstanding compared to other genotypes.											
(**c**)											
**Genotype**	***Crop 1***	***Crop 2***	***Crop 3***	Mean *	***CV (%)***	**Genotype**	***Crop 1***	***Crop 2***	***Crop 3***	Mean *	***CV (%)***
**Verdim R**	**1.53 ± 0.03 ^a^**	**1.41 ± 0.03 ^b^**	**1.39 ± 0.03 ^b^**	**1.44 ± 0.08**	**5.30**	Z38	1.13 ± 0.03 ^b^	1.25 ± 0.02 ^a^	1.15 ± 0.03 ^b^	1.17 ± 0.06	5.48
B01	0.93 ± 0.03 ^b^	0.88 ± 0.03 ^b^	1.22 ± 0.02 ^a^	1.01 ± 0.19	18.47	**Z18**	**1.55 ± 0.02 ^c^**	**1.72 ± 0.02 ^a^**	**1.66 ± 0.02 ^b^**	**1.64 ± 0.08**	**5.06**
Bicudo	1.30 ± 0.01 ^b^	1.28 ± 0.02 ^b^	1.52 ± 0.02 ^a^	1.37 ± 0.13	9.63	Z17	1.20 ± 0.03 ^b^	1.24 ± 0.02 ^a^	1.16 ± 0.02 ^c^	1.20 ± 0.04	3.31
**Alecrim**	**1.54 ± 0.04 ^c^**	**1.64 ± 0.02 ^b^**	**1.75 ± 0.03 ^a^**	**1.65 ± 0.11**	**6.48**	Z21	1.65 ± 0.03 ^a^	1.39 ± 0.02 ^b^	1.11 ± 0.08 ^c^	1.38 ± 0.27	19.41
**700**	**1.69 ± 0.03 ^c^**	**1.77 ± 0.02 ^b^**	**1.84 ± 0.02 ^a^**	**1.77 ± 0.07**	**4.16**	Z36	1.23 ± 0.02 ^b^	1.21 ± 0.04 ^b^	1.32 ± 0.03 ^a^	1.25 ± 0.06	4.83
CH1	0.97 ± 0.02 ^c^	1.20 ± 0.01 ^a^	1.14 ± 0.02 ^b^	1.10 ± 0.12	10.84	Ouro negro	1.20 ± 0.02 ^a^	1.07 ± 0.02 ^c^	1.15 ± 0.03 ^b^	1.14 ± 0.06	5.68
Imbigudinho	1.17 ± 0.01 ^b^	1.06 ± 0.01 ^c^	1.22 ± 0.02 ^a^	1.15 ± 0.08	7.27	18	1.00 ± 0.04 ^a^	0.86 ± 0.03 ^b^	1.04 ± 0.03 ^a^	0.97 ± 0.09	9.70
AT	1.13 ± 0.02 ^b^	1.31 ± 0.02 ^a^	1.10 ± 0.01 ^b^	1.18 ± 0.11	9.50	Tardio C	1.02 ± 0.02 ^b^	1.04 ± 0.02 ^b^	1.10 ± 0.02 ^a^	1.05 ± 0.04	4.08
Graudão HP	1.08 ± 0.02 ^b^	1.12 ± 0.01 ^b^	1.20 ± 0.03 ^a^	1.13 ± 0.06	5.44	**A1**	**1.69 ± 0.04 ^c^**	**1.72 ± 0.02 ^b^**	**1.76 ± 0.09 ^a^**	**1.73 ± 0.04**	**2.05**
**Valcir P**	**1.35 ± 0.03 ^a^**	**1.22 ± 0.03 ^b^**	**1.32 ± 0.02 ^a^**	**1.30 ± 0.07**	**5.05**	**Cheique**	**1.50 ± 0.02 ^c^**	**1.54 ± 0.01 ^b^**	**1.69 ± 0.02 ^a^**	**1.57 ± 0.10**	**6.44**
**Beira Rio 8**	**1.50 ± 0.03 ^c^**	**1.63 ± 0.07 ^b^**	**1.75 ± 0.02 ^a^**	**1.63 ± 0.12**	**7.56**	**P2**	**1.69 ± 0.02 ^a^**	**1.69 ± 0.02 ^a^**	**1.43 ± 0.07 ^b^**	**1.60 ± 0.15**	**9.47**
Tardio V	0.83 ± 0.02 ^c^	1.01 ± 0.02 ^b^	1.19 ± 0.02 ^a^	1.01 ± 0.18	17.37	**Emcapa 02**	**1.76 ± 0.03 ^a^**	**1.65 ± 0.02 ^b^**	**1.69 ± 0.02 ^b^**	**1.70 ± 0.05**	**3.10**
**AP**	**1.52 ± 0.02 ^b^**	**1.61 ± 0.04 ^a^**	**1.44 ± 0.01 ^c^**	**1.52 ± 0.08**	**5.50**	Emcapa 153	1.24 ± 0.02 ^b^	1.29 ± 0.02 ^a^	1.19 ± 0.02 ^c^	1.24 ± 0.05	4.31
**L80**	**1.55 ± 0.03 ^c^**	**1.76 ± 0.03 ^a^**	**1.60 ± 0.02 ^b^**	**1.64 ± 0.11**	**6.62**	P1	0.95 ± 0.01 ^c^	1.10 ± 0.01 ^b^	1.17 ± 0.02 ^a^	1.07 ± 0.11	10.36
Bamburral	1.09 ± 0.02 ^c^	1.23 ± 0.02 ^b^	1.35 ± 0.03 ^a^	1.23 ± 0.13	10.48	LB1	1.33 ± 0.03 ^b^	1.45 ± 0.04 ^a^	1.33 ± 0.01 ^b^	1.37 ± 0.07	5.34
Pirata	1.02 ± 0.02 ^c^	1.09 ± 0.03 ^b^	1.24 ± 0.01 ^a^	1.12 ± 0.11	10.21	**122**	**1.73 ± 0.04 ^a^**	**1.64 ± 0.04 ^b^**	**1.74 ± 0.06 ^a^**	**1.70 ± 0.06**	**3.30**
Peneirão	0.98 ± 0.01 ^c^	1.12 ± 0.03 ^a^	1.10 ± 0.02 ^b^	1.07 ± 0.09	7.30	Verdim D	1.43 ± 0.03 ^a^	1.41 ± 0.03 ^a^	1.44 ± 0.01 ^a^	1.42 ± 0.02	1.15
Z39	1.21 ± 0.01 ^b^	1.36 ± 0.02 ^a^	1.36 ± 0.03 ^a^	1.31 ± 0.08	6.64	Emcapa 143	0.77 ± 0.02 ^c^	0.85 ± 0.02 ^b^	1.11 ± 0.02 ^a^	0.91 ± 0.17	19.04
Z35	0.92 ± 0.01 ^b^	0.93 ± 0.02 ^b^	1.06 ± 0.03 ^a^	0.97 ± 0.08	8.10	Ouro negro 1	1.28 ± 0.05 ^c^	1.38 ± 0.02 ^b^	1.56 ± 0.01 ^a^	1.41 ± 0.14	10.12
Z40	0.96 ± 0.02 ^a^	0.91 ± 0.02 ^b^	0.74 ± 0.03 ^c^	0.87 ± 0.12	13.41	Ouro negro 2	1.03 ± 0.03 ^b^	0.97 ± 0.01 ^c^	1.35 ± 0.02 ^a^	1.12 ± 0.21	18.52
Z29	1.10 ± 0.03 ^a^	0.98 ± 0.01 ^b^	1.01 ± 0.02 ^b^	1.03 ± 0.06	5.85	**Clementino**	**1.73 ± 0.02 ^b^**	**1.81 ± 0.01 ^a^**	**1.60 ± 0.03 ^c^**	**1.71 ± 0.11**	**6.31**
Note (**c**): * Average trigonelline contents of the three consecutive crops (2018, 2019, and 2020). CV: coefficient of variation. Different letters for the same genotype indicate statistical differences between crops by ANOVA (*p* < 0.05). Values in bold were consistently outstanding compared to other genotypes.											

### 2.4. Correlations Among Bioactive Compounds in the Different Parts of the Plant

Due to their metabolic and physiological origin, many chemical compounds can correlate with each other. In addition to being present in the same cell compartment [50], caffeine forms complexes with CGA, specifically with CQA [51]. Considering all genotypes, this study observed a discrete correlation between the mean contents of CGA and caffeine in the seeds (r: 0.72, *p* = 0.003). Still in the seeds, a tendency was observed for positive correlations between the contents of soluble solids and caffeine (r: 0.64, *p* = 0.0299), trigonelline (r: 0.64, *p* = 0.0324) and total CGA (r: 0.78, *p* < 0.001), which was expected, since the total content of soluble solids is composed mainly of sugars, CGA, caffeine and trigonelline, and other minor substances [52]. Additional correlations were observed between the contents of FQA and diCQA in husks (r: 0.94, *p* < 0.001) and leaves (r: 0.69, *p* < 0.001). The correlation results were similar when we considered each crop separately and all crops together. No other relevant correlations were found among the bioactive compounds in the different parts of the studied genotypes.

### 2.5. Selection of Promising Genotypes

As aforementioned, the contents of bioactive compounds may vary according to factors such as genetics (species and cultivar), physiological, and edaphoclimatic conditions and cultivation practices, especially nitrogen fertilization [20,31,40]. In the present work, there has been no change in the fertilization practices during the three consecutive crops evaluated. Therefore, the variations in the content of bioactive compounds and soluble solids among the different genotypes studied were more significant and more important than those among crops. Different from arabica coffees, this large variability can be expected in canefora coffees given the polygenic determinism and heterozygosity, with hereditary transmission, in which the expression of characteristics depends on their meiotic behavior. *Canephora plants* are characterized by having diploid cells (2*n* = 2) and 22 chromosomes, and their reproduction occurs through allogamy or cross-pollination, with the participation of two gametes [53,54]. The reproductive self-incompatibility of the canefora plants and the consequent inability of self-fertilization or pollination between coffee plants with similar reproductive gamete organization leads to greater genetic variability [53,54]. This leads to a greater diversity of characteristics, which makes production heterogeneous, with low standardization of important characteristics for production and commercialization, such as fruit ripening time, shape, seed size, resistance to pests, and climatic variations [55] and therefore the lack of standardization in the chemical compositions of these genotypes can be also expected.

Charrier and Berthaud [56] observed that the average caffeine content of each progeny is close to the arithmetic mean of the parents’ content, and the variability of the offspring is associated with the degree of heterozygosity of these parents, consequently, with their mode of reproduction. Therefore, caffeine content shows less variability in the arabica species (autogamous) when compared to those in the highly heterozygous (allogamic) canefora species, hence the importance of propagation by cuttings when desirable characteristics are observed in each canefora plant.

Based on the total contents of CGA and soluble solids in the seeds of five selected genotypes (Graudão HP, Tardio V, Z 37, Tardio C, and Emcapa 02), the registration of a new cultivar was granted in 2020 by the Brazilian Ministry of Agriculture and Livestock under the name of “Salutar”, with mean soluble solids of 4.53 °Brix and CGA contents of 9.02 g/100 g db [57]. Greater vigor and resistance to practices and diseases and satisfactory productivity (about 5 tons/ha) were also considered when selecting the five genotypes. In Brazil, these genotypes can be cultivated in climatic conditions similar to those in Nova Venécia, where they were grown, for example, and in other locations such as the Southern region of Bahia and Eastern region of Minas Gerais, at altitudes below 600 m. However, with global warming different regions and altitudes worldwide may be a possibility. In addition to the higher amount of CGA and, therefore, higher potential antioxidants and anti-inflammatory activities [17,19], the high content of soluble solids is an important factor in meeting the market demand for soluble coffee production. Data from the Brazilian Association of the Soluble Coffee Industry (ABICS) [58] revealed that in 2024, Brazil was the world’s largest producer of soluble coffee, exporting over 94 thousand tons. The main destinations were the United States, Indonesia, Russia, and Argentina.

Regular consumption of caffeine in moderate doses (up to 400 mg/day or equivalent to 3-4 100 mL cups of coffee/day) has been associated with the prevention of chronic and neurodegenerative diseases like diabetes, cancer, Parkinson’s, and Alzheimer’s [59]. For this reason, the genotypes that present high contents of caffeine (B01, Alecrim, Imbigudinho, Beira Rio 8, Tardio C, Cheique, and P2–2.14-2.63 g/100 g db) can be explored and valued for beverages with higher contents of this compound. Differently from what is expected when we think of canefora genotypes, lower caffeine contents were observed in genotype L80 (1.21–1.42 g/100 g db), which can also be interesting for soluble coffee with lower caffeine content than those traditionally containing 100% conilon seeds.

## 3. Materials and Methods

### 3.1. Samples and Experiment Set Up

**Figure 2:**
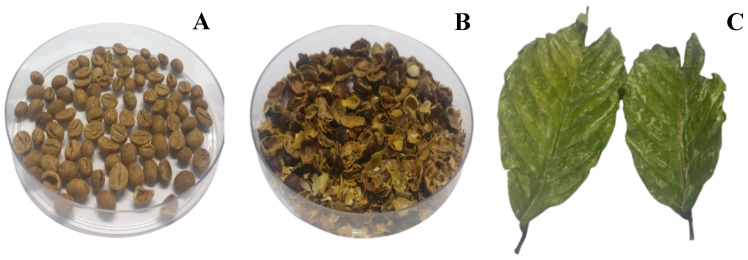
Dried green seeds (**A**), husks (**B**) and leaves (**C**) of new genotypes (*n* = 42) of *C. canephora* originating from the conilon cultivar.

Promising plants of *C. canephora* cv. conilon (*n* = 42) were selected by different coffee growers based on higher productivity, resistance to climate changes and pests, and beverage quality. The genotypes were vegetatively replicated by cuttings (clonal propagation) and cultivated in an experimental area as a “competition trial”. The plant parts evaluated included green seeds, husks, and leaves from three consecutive crops (2018, 2019, and 2020) (Figure 2).

**Figure 3:**
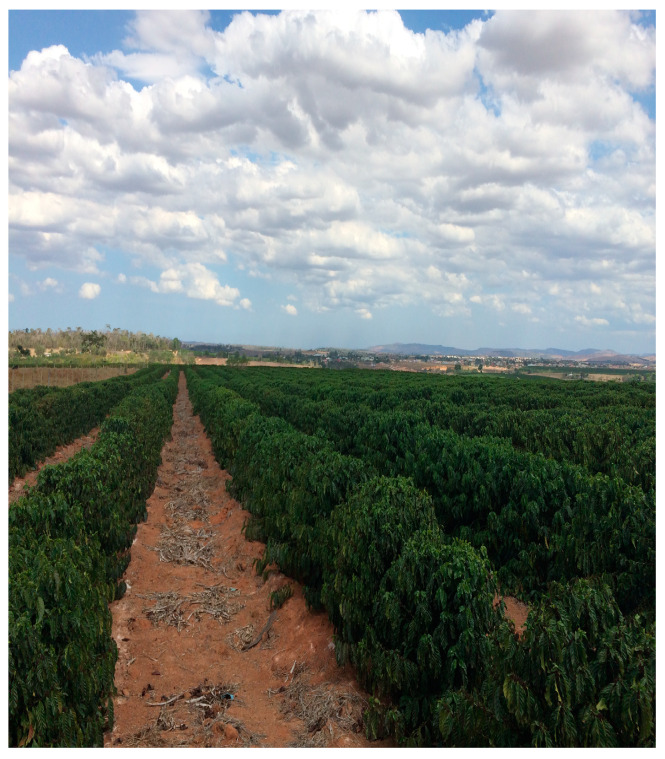
Four years old *C. canephora* conilon plants in the farm located in Nova Venécia, Espirito Santo, Brazil, where the experiment was set up.

The experiment was set up in Nova Venécia, Espírito Santo, Brazil, on a private property located at latitude 18°39′43″ S and longitude 40°25′52″ W, 199 m of altitude, and an annual average temperature of 23 °C. The region has a tropical climate, characterized by a hot and humid summer and dry winter, classified as Aw, according to the Köppen classification [60]. The design used to plant the genotypes was a randomized block design with three field replications, each replicating seven plants. Fertilization was carried out according to soil analysis, and pruning was carried out to control excessive branches, maintaining the standard of 12,000 to 15,000 stems per hectare. In all experimental years, manual weeding (threshing at the fertilization site), mechanized, and chemical weeding were carried out. Nutrients, insecticides, and fungicides were applied when necessary, during the years of study to all genotypes equally, in controlled quantities, although there was no severe attack from the main coffee pests and diseases, and the plants remained vigorous and with good foliage. The experimental area was irrigated all year long with a drip irrigation system. During the years of evaluation, the adaptation of genotypes to the cultivation conditions, along with performance in growth and production, were evaluated (Figure 3).

### 3.2. Harvest and Post-Harvest

Samples were collected for analysis when the plants were 4, 5, and 6 years old (2018, 2019, and 2020 crops). The coffee fruits were harvested manually at their ideal ripening stage (“cherry” stage) in the morning. They were dried in a forced circulation oven (ShelLab^®^, SMO14-2) at 50 °C, for 7 days (dry method). Subsequently, the fruits were crushed, and the husks and seeds (without defects) were separated. Regarding the leaves, adult (the third pairs in the branches) samples were collected manually and dried in the same forced circulation oven (50 °C) for 2 days. Cultivation and post-harvest processing conditions were the same in the three crops.

### 3.3. Water Content

In order to express the contents of bioactive compounds on a dry weight basis (db), the water content of the dried flowers (expressed as percentage) was determined using a MX-50 moisture analyzer (A&D Company, Tokyo, Japan).

### 3.4. Soluble Solids of Green Seeds

The seeds aqueous extracts were prepared at 10% (weight to volume). They were then filtered through Whatman paper number 1. Soluble solids were determined using a digital refractometer (Atago^®^, PAL-1, Tokyo, Japan), according to the adaptation of the method number 15.034 of the AOAC [61]. The results were expressed in °Brix.

### 3.5. Methanolic Extraction and Analysis of Bioactive Compounds

Extractions were performed in triplicate, according to a modification of the method by Trugo and Macrae [62] described in detail in Farah et al. [63]. Half a gram of green seed, husk, or leaf was suspended in 60 mL of methanol 40% and shaken at room temperature for 15 min at 300 rpm. The mixture was filtered through filter paper (Whatman n°1) and washed with 30 mL of water. The extracts were clarified for precipitation of proteins and other high molecular weight compounds with 1 mL of each of Carrez solutions (zinc acetate 1.0 M and potassium ferricyanide 0.3 M) for analyses of caffeine and phenolic compounds and with lead acetate (60%) for trigonelline. Following, the volume was made up to 100 mL with water, the mixture was shaken for 5 s and left to stand for 10 min. The colloidal precipitation was then filtered (Whatman n°1), and the filtrate was used directly for chromatographic analysis. For analyses of bioactive compounds, a HPLC-DAD-reverse phase system with a high-precision pump (Shimadzu^®^ model LC-10-AD, Kyoto, Japan), a DAD detector (Shimadzu model LC-10-AD), and a C18 column (Phenomenex, Torrance, CA, USA, Luna^®^, 5 µm, 250 × 4.6 mm^2^) was used. Chromatographic data was recorded and integrated using the LC Solution computer software (Shimadzu). For CGA analysis, a gradient was performed using 0.3% formic acid (eluent A) and methanol 100% (eluent B) as mobile phase at a 1 mL/min flow rate. The injection volume was 50 μL, and DAD operated at 257–340 nm [63]. For identification, UV spectra and LC-MS (LCMS 2010, Shimadzu, Kyoto, Japan) were used, in addition to standards (see below). The quantification of all CGA was performed using the area of 5-caffeoylquinic acid (5-CQA) standard combined with the molar extinction coefficients of CGA of their direct precursors, as thoroughly explained in Farah et al. [63]. The limit of quantification (LOQ) for CGA and phenolic acids was 2 µg/100 g. Regarding standards, 5-CQA, phenolic acids (cafeic, ferulic, *p*-coumaric, gallic, benzoic, 3,4-dihydroxibenzoic), rutin, quercetin, and kaempferol were purchased from Sigma-Aldrich (St Louis, MO, USA). A mixture of 3-caffeoylquinic (3-CQA) and 4-caffeoylquinic (4-CQA) was prepared from 5-CQA, using the isomerization method of Trugo and Macrae [62]. For dicafeoylquinic acids (diCQA), a mixture of 3,4-diCQA; 3,5-diCQA; and 4,5-diCQA from Carl Roth (Karlsruhe, Germany). Standard solutions of diCQA were used for peak confirmation. Feruloylquinic acids (FQA) were obtained by hydrolysis of 3-feruloylquinide (3-FQL) and 4-feruloylquinide (4-FQL) in 50% aqueous tetrahydro-furan [64]. The present work used the IUPAC numbering system for CGA identification. An isocratic system was used to analyze caffeine and trigonelline. The mobile phase consisted of 40% methanol for caffeine and 5% methanol for trigonelline. DAD operated at 272 nm for caffeine and 264 nm for trigonelline [63]. LOQ for both caffeine and trigonelline under these conditions was 3 µg/100 g. Standards of caffeine and trigonelline (as trigonelline hydrochloride) were purchased from Sigma-Aldrich.

### 3.6. Statistical Analysis

Chromatographic analysis results were presented as mean (dry basis—db) ± standard deviation, and the Coeficient of Variation (CV) was determined for all genotypes. Results were statistically tested for differences and comparisons among crops and genotypes, using analysis of variance (ANOVA), followed by the Fisher test (Statistica^®^, version 13.4.0.14, New York, NY, USA). Pearson’s correlations were determined considering the contents of bioactive compounds in the parts of coffee plants analyzed. Differences were considered when *p* < 0.05. Additionally, multivariate analysis was used to produce heat maps (Statistica^®^, version 13.4.0.14, United States).

## 4. Concluding Remarks

The contents of caffeine, trigonelline, and total CGA varied considerably among the green seeds, husks, and leaves of the forty-two studied genotypes of *C. canephora* cv. conilon. CGA contents (g/100 g, db) in seeds, husks, and leaves ranged between 3.71 and 9.71, 0.43 and 1.65, and 0.80 and 2.22, respectively. Caffeine contents (g/100 g, db) in seeds, husks, and leaves ranged between 1.21 and 2.63, 0.13 and 0.84, and 0.33 and 2.01, respectively. Trigonelline contents (g/100 g, db) ranged between 0.83 and 1.12, 0.59 and 1.24, and 0.74 and 1.84 , respectively. Variation among the three crops was higher for CGA. Nevertheless, some of the genotypes showed higher contents of bioactive compounds than others, demonstrating promising potential as a material for brewing puposes or for extraction of bioactive compounds. These are good candidates for cultivar registration, with possibilities to meet various market demands in the food and pharmaceutical industries.

The caffeine in industrialized products comes from the extraction of this compound naturally present in plant materials. Coffee seeds are the leading suppliers through the decaffeination process carried out before roasting. Currently, the demand for caffeine in the food and pharmaceutical industries is higher than the amount of decaffeinated coffee produced, and new sources of caffeine are needed [65]. Coffee byproducts with high content of caffeine are candidates to help fulfill this need. Therefore, knowledge of the bioactive profile of coffee byproducts is important for their full use in reducing waste and environmental damage generated by coffee processing, whether in the form of beverages or in the food and pharmaceutical industries.

Considering the importance of coffee as a food product for human consumption in the world market and all the recent discoveries on its positive health effects, research is essential for the selection of promising plants, favoring increased production, cup quality, resistance to pests, and contents of bioactive compounds, and allowing greater utilization of all byproducts, improving economic returns and the quality of human life, in accordance with the 2030 United Nations Agenda. Furthermore, considering current trends and global warming, conilon coffee emerges as an alternative for the future of coffee farming when combined with currently existing and ever-changing post-harvesting methods. Once the genotypes are selected, the method of clonal propagation by cuttings maintains the genetic characteristics of the mother plant and appears to be the best way to establish cultivars with greater homogenization of the future crops in terms of their physical and chemical characteristics.

## Figures and Tables

**Table 1:** Water content (%) of green seeds, husks, and leaves of selected *C. canephora* cv. conilon genotypes, from three consecutive crops.

	*Seeds*			*Husks*			*Leaves*		
Genotype	Crop 1	Crop 2	Crop 3	Crop 1	Crop 2	Crop 3	Crop 1	Crop 2	Crop 3
Verdim R	8.9 ± 0.2	8.4 ± 0.2	8.54 ± 0.3	11.2 ± 0.2	11.0 ± 0.2	11.1 ± 0.3	10.1 ± 0.2	9.6 ± 0.1	9.8 ± 0.1
B01	8.7 ± 0.2	8.5 ± 0.2	8.6 ± 0.2	10.3 ± 0.2	10.6 ± 0.1	10.6 ± 0.1	10.3 ± 0.1	10.5 ± 0.2	10.2 ± 0.2
Bicudo	8.6 ± 0.1	8.4 ± 0.2	8.4 ± 0.1	11.1 ± 0.3	11.0 ± 0.1	10.9 ± 0.3	10.4 ± 0.2	10.1 ± 0.1	10.0 ± 0.2
Alecrim	9.0 ± 0.1	8.7 ± 0.2	8.8 ± 0.1	11.1 ± 0.3	10.7 ± 0.0	10.9 ± 0.2	9.9 ± 0.1	9.7 ± 0.1	9.5 ± 0.2
700	9.1 ± 0.0	9.0 ± 0.1	9.2 ± 0.2	11.2 ± 0.3	10.6 ± 0.0	10.5 ± 0.1	10.8 ± 0.1	10.5 ± 0.1	10.1 ± 0.3
CH1	8.9 ± 0.2	9.0 ± 0.2	8.9 ± 0.1	11.3 ± 0.2	10.7 ± 0.0	10.9 ± 0.2	11.0 ± 0.2	10.3 ± 0.1	10.2 ± 0.2
Imbigudinho	8.7 ± 0.1	9.0 ± 0.3	9.2 ± 0.3	11.2 ± 0.2	11.2 ± 0.1	11.0 ± 0.1	11.0 ± 0.3	10.2 ± 0.1	10.1 ± 0.3
AT	8.8 ± 0.1	8.5 ± 0.2	8.7 ± 0.3	11.8 ± 0.2	11.6 ± 0.2	11.4 ± 0.2	10.1 ± 0.2	10.0 ± 0.2	10.5 ± 0.3
Graudão HP	9.1 ± 0.1	9.2 ± 0.3	9.0 ± 0.2	11.2 ± 0.1	10.4 ± 0.2	10.2 ± 0.2	10.1 ± 0.2	10.0 ± 0.0	9.7 ± 0.4
Valcir P	9.2 ± 0.3	9.0 ± 0.1	8.9 ± 0.3	11.2 ± 0.1	10.5 ± 0.2	10.4 ± 0.3	10.4 ± 0.1	10.1 ± 0.0	10.2 ± 0.0
Beira Rio 8	8.6 ± 0.1	8.3 ± 0.1	8.5 ± 0.1	11.4 ± 0.1	10.3 ± 0.2	10.2 ± 0.3	10.2 ± 0.1	10.1 ± 0.2	10.0 ± 0.3
Tardio V	8.7 ± 0.1	8.4 ± 0.1	8.3 ± 0.1	11.3 ± 0.1	11.3 ± 0.1	11.0 ± 0.2	10.6 ± 0.1	10.8 ± 0.1	10.5 ± 0.2
AP	8.8 ± 0.0	8.9 ± 0.1	9.0 ± 0.1	10.8 ± 0.2	11.3 ± 0.2	11.5 ± 0.2	11.1 ± 0.3	10.7 ± 0.1	10.6 ± 0.1
L80	8.9 ± 0.0	8.9 ± 0.1	9.2 ± 0.3	11.3 ± 0.2	11.5 ± 0.3	11.1 ± 0.2	10.7 ± 0.3	10.5 ± 0.1	10.5 ± 0.1
Bamburral	9.2 ± 0.1	9.0 ± 0.0	9.2 ± 0.0	10.7 ± 0.1	11.3 ± 0.2	11.0 ± 0.3	10.8 ± 0.2	10.9 ± 0.2	11.0 ± 0.1
Pirata	9.5 ± 0.1	9.4 ± 0.3	9.3 ± 0.2	12.7 ± 0.2	12.0 ± 0.2	12.5 ± 0.2	9.9 ± 0.1	10.0 ± 0.0	10.3 ± 0.2
Peneirão	8.4 ± 0.1	8.7 ± 0.1	8.9 ± 0.2	10.7 ± 0.1	11.4 ± 0.2	11.0 ± 0.1	9.9 ± 0.1	10.0 ± 0.1	10.2 ± 0.2
Z39	8.2 ± 0.1	8.0 ± 0.2	8.4 ± 0.3	11.4 ± 0.1	11.0 ± 0.1	11.2 ± 0.3	10.1 ± 0.0	10.4 ± 0.1	10.5 ± 0.3
Z35	8.0 ± 0.0	8.2 ± 0.1	8.0 ± 0.4	10.9 ± 0.0	10.8 ± 0.1	10.9 ± 0.2	10.5 ± 0.0	10.3 ± 0.1	10.1 ± 0.4
Z40	8.5 ± 0.2	8.7 ± 0.1	8.5 ± 0.2	10.7 ± 0.0	11.1 ± 0.2	11.0 ± 0.3	11.2 ± 0.0	11.0 ± 0.3	11.1 ± 0.2
Z29	8.6 ± 0.2	8.9 ± 0.1	9.0 ± 0.4	11.4 ± 0.1	11.0 ± 0.1	11.2 ± 0.3	10.1 ± 0.0	10.4 ± 0.1	10.5 ± 0.3
Z38	8.8 ± 0.1	9.1 ± 0.2	9.2 ± 0.3	11.2 ± 0.1	11.7 ± 0.2	11.1 ± 0.4	10.9 ± 0.2	11.0 ± 0.2	11.2 ± 0.1
Z18	9.2 ± 0.2	9.1 ± 0.1	9.3 ± 0.3	11.3 ± 0.1	11.5 ± 0.2	11.2 ± 0.1	10.5 ± 0.3	10.7 ± 0.2	10.4 ± 0.1
Z37	8.2 ± 0.1	8.4 ± 0.1	8.5 ± 0.3	11.2 ± 0.1	11.6 ± 0.2	11.4 ± 0.0	10.1 ± 0.2	10.3 ± 0.1	10.3 ± 0.2
Z21	9.1 ± 0.1	9.1 ± 0.2	9.0 ± 0.4	10.8 ± 0.0	11.3 ± 0.3	11.2 ± 0.4	11.0 ± 0.4	10.7 ± 0.1	11.0 ± 0.2
Z36	8.3 ± 0.1	8.3 ± 0.2	8.6 ± 0.2	10.7 ± 0.2	11.2 ± 0.2	11.1 ± 0.2	9.8 ± 0.2	10.0 ± 0.0	10.2 ± 0.1
Ouro negro	8.2 ± 0.1	8.2 ± 0.1	8.3 ± 0.3	12.7 ± 0.2	12.5 ± 0.3	12.2 ± 0.4	9.5 ± 0.2	10.2 ± 0.1	10.3 ± 0.1
18	8.4 ± 0.2	8.4 ± 0.2	8.5 ± 0.4	11.0 ± 0.1	11.3 ± 0.1	11.1 ± 0.3	9.9 ± 0.2	10.2 ± 0.2	10.3 ± 0.2
Tardio C	9.0 ± 0.2	9.1 ± 0.3	9.2 ± 0.4	10.8 ± 0.1	11.0 ± 0.1	11.3 ± 0.3	10.3 ± 0.1	10.5 ± 0.1	10.1 ± 0.2
A1	8.5 ± 0.1	8.5 ± 0.3	8.3 ± 0.2	13.4 ± 0.2	12.5 ± 0.1	12.3 ± 0.4	10.1 ± 0.1	10.1 ± 0.1	10.2 ± 0.1
Cheique	9.2 ± 0.1	9.0 ± 0.0	9.4 ± 0.2	10.9 ± 0.1	11.1 ± 0.1	10.8 ± 0.2	10.4 ± 0.1	10.4 ± 0.1	10.5 ± 0.1
P2	9.3 ± 0.2	9.4 ± 0.4	9.0 ± 0.4	10.6 ± 0.1	10.7 ± 0.1	10.5 ± 0.4	10.6 ± 0.2	10.5 ± 0.1	10.4 ± 0.2
Emcapa 02	8.7 ± 0.0	8.5 ± 0.2	8.4 ± 0.3	10.8 ± 0.2	10.9 ± 0.2	11.1 ± 0.1	10.2 ± 0.2	10.2 ± 0.1	10.1 ± 0.3
Emcapa 153	8.5 ± 0.0	8.6 ± 0.1	8.3 ± 0.3	10.6 ± 0.2	10.4 ± 0.2	10.5 ± 0.3	10.4 ± 0.1	10.4 ± 0.1	10.2 ± 0.1
P1	8.2 ± 0.1	8.0 ± 0.2	8.5 ± 0.3	10.9 ± 0.2	11.2 ± 0.2	11.4 ± 0.2	9.8 ± 0.0	10.1 ± 0.1	10.3 ± 0.1
LB1	8.1 ± 0.0	8.1 ± 0.2	8.5 ± 0.4	10.7 ± 0.1	10.5 ± 0.1	10.3 ± 0.2	10.9 ± 0.1	10.3 ± 0.1	10.4 ± 0.3
122	9.2 ± 0.2	8.7 ± 0.0	9.2 ± 0.3	11.1 ± 0.1	11.6 ± 0.3	11.1 ± 0.2	11.1 ± 0.1	11.0 ± 0.1	11.1 ± 0.2
Verdim D	9.2 ± 0.2	9.2 ± 0.3	9.3 ± 0.3	11.1 ± 0.1	11.6 ± 0.3	11.5 ± 0.2	10.9 ± 0.1	11.1 ± 0.1	11.2 ± 0.1
Emcapa 143	8.5 ± 0.2	8.6 ± 0.1	8.5 ± 0.3	10.6 ± 0.1	10.9 ± 0.3	10.5 ± 0.3	10.3 ± 0.1	10.4 ± 0.0	10.4 ± 0.3
Ouro negro 1	9.0 ± 0.1	9.2 ± 0.0	9.0 ± 0.4	11.4 ± 0.1	11.5 ± 0.1	11.3 ± 0.0	10.8 ± 0.1	10.7 ± 0.0	10.5 ± 0.3
Ouro negro 2	8.4 ± 0.1	8.7 ± 0.1	9.0 ± 0.3	11.3 ± 0.1	11.5 ± 0.1	11.1 ± 0.2	11.0 ± 0.2	10.7 ± 0.1	10.8 ± 0.1
Clementino	8.7 ± 0.1	8.5 ± 0.0	8.8 ± 0.3	12.3 ± 0.2	12.0 ± 0.2	11.7 ± 0.4	11.2 ± 0.2	10.7 ± 0.1	11.0 ± 0.3

**Table 2:** Soluble solids content (°Brix) of green seeds of selected *C. canephora* cv. conilon genotypes, from three consecutive crops.

Genotype	*Crop 1*	*Crop 2*	*Crop 3*	Mean *	*CV (%)*	Genotype	*Crop 1*	*Crop 2*	*Crop 3*	Mean *	*CV (%)*
Verdim R	3.6 ± 0.2 ^a^	3.8 ± 0.1 ^a^	3.3 ± 0.2 ^b^	3.6 ± 0.3	7.28	Z38	3.9 ± 0.0 ^b^	4.0 ± 0.1 ^a^	3.5 ± 0.1 ^c^	3.8 ± 0.3	6.77
**B01**	**4.1 ± 0.1 ^b^**	**4.4 ± 0.1 ^a^**	**4.1 ± 0.1 ^b^**	**4.2 ± 0.2**	**4.12**	Z18	3.8 ± 0.0 ^a^	4.1 ± 0.2 ^a^	4.1 ± 0.2 ^a^	4.0 ± 0.2	4.78
Bicudo	4.1 ± 0.1 ^a^	4.3 ± 0.0 ^a^	3.9 ± 0.2 ^b^	4.1 ± 0.3	5.33	**Z17**	**4.2 ± 0.1 ^b^**	**4.6 ± 0.2 ^a^**	**4.5 ± 0.1 ^a^**	**4.5 ± 0.2**	**4.66**
Alecrim	4.0 ± 0.0 ^a^	4.1 ± 0.1 ^a^	3.6 ± 0.2 ^b^	3.9 ± 0.3	7.27	Z21	3.9 ± 0.1 ^a^	3.8 ± 0.1 ^a^	3.9 ± 0.1 ^a^	3.9 ± 0.1	1.31
700	3.6 ± 0.1 ^a^	3.7 ± 0.0 ^a^	3.5 ± 0.2 ^a^	3.6 ± 0.1	2.45	**Z36**	**4.5 ± 0.1 ^a^**	**4.4 ± 0.0 ^a^**	**4.5 ± 0.1 ^a^**	**4.5 ± 0.1**	**1.55**
CH1	3.2 ± 0.0 ^b^	3.5 ± 0.1 ^a^	3.1 ± 0.1 ^b^	3.3 ± 0.2	7.07	Ouro negro	4.0 ± 0.1 ^a^	4.2 ± 0.0 ^a^	3.9 ± 0.3 ^a^	4.1 ± 0.1	3.32
Imbigudinho	3.5 ± 0.1 ^a^	3.7 ± 0.1 ^a^	3.5 ± 0.2 ^a^	3.5 ± 0.1	3.02	18	3.5 ± 0.1 ^b^	3.9 ± 0.0 ^a^	3.9 ± 0.1 ^a^	3.8 ± 0.2	6.13
AT	3.2 ± 0.1 ^a^	3.3 ± 0.0 ^a^	2.9 ± 0.1 ^b^	3.1 ± 0.2	7.45	**Tardio C**	**4.4 ± 0.1 ^b^**	**4.9 ± 0.0 ^a^**	**4.9 ± 0.0 ^a^**	**4.7 ± 0.3**	**6.52**
**Graudão HP**	**4.2 ± 0.1 ^b^**	**4.7 ± 0.1 ^a^**	**4.3 ± 0.2 ^b^**	**4.4 ± 0.3**	**5.97**	A1	4.3 ± 0.1 ^a^	3.7 ± 0.1 ^b^	3.8 ± 0.0 ^b^	3.9 ± 0.3	7.85
Valcir P	3.8 ± 0.0 ^b^	4.2 ± 0.2 ^a^	3.9 ± 0.1 ^ab^	4.0 ± 0.2	4.84	Cheique	4.1 ± 0.1 ^a^	4.2 ± 0.0 ^a^	3.7 ± 0.2 ^b^	4.0 ± 0.2	6.01
Beira Rio 8	3.8 ± 0.1 ^b^	4.1 ± 0.1 ^a^	3.9 ± 0.0 ^ab^	3.9 ± 0.2	4.28	P2	3.9 ± 0.2 ^b^	4.1 ± 0.0 ^ab^	4.3 ± 0.1 ^a^	4.1 ± 0.2	4.47
**Tardio V**	**4.4 ± 0.1 ^a^**	**4.4 ± 0.1 ^a^**	**4.3 ± 0.1 ^a^**	**4.4 ± 0.1**	**1.93**	**Emcapa 02**	**4.7 ± 0.1 ^b^**	**5.0 ± 0.2 ^a^**	**4.4 ± 0.1 ^b^**	**4.7 ± 0.3**	**6.42**
**AP**	**4.2 ± 0.2 ^a^**	**4.3 ± 0.1 ^a^**	**4.0 ± 0.1 ^b^**	**4.2 ± 0.2**	**4.54**	Emcapa 153	3.7 ± 0.1 ^b^	3.8 ± 0.1 ^a^	3.6 ± 0.0 ^b^	3.7 ± 0.1	3.25
L80	3.4 ± 0.1 ^a^	3.3 ± 0.1 ^a^	3.0 ± 0.2 ^b^	3.2 ± 0.2	5.84	P1	4.0 ± 0.2 ^a^	3.7 ± 0.1 ^a^	3.9 ± 0.2 ^a^	3.9 ± 0.2	3.92
Bamburral	3.4 ± 0.1 ^a^	3.3 ± 0.0 ^b^	3.0 ± 0.2 ^c^	3.2 ± 0.2	7.43	LB1	3.4 ± 0.2 ^ab^	3.2 ± 0.1 ^b^	3.7 ± 0.2 ^a^	3.5 ± 0.3	7.26
Pirata	3.8 ± 0.1 ^a^	3.9 ± 0.0 ^a^	3.5 ± 0.3 ^a^	3.7 ± 0.2	4.97	**122**	**4.1 ± 0.2 ^a^**	**4.0 ± 0.0 ^a^**	**4.0 ± 0.3 ^a^**	**4.0 ± 0.1**	**2.19**
**Peneirão**	**4.2 ± 0.1 ^b^**	**4.6 ± 0.1 ^a^**	**4.1 ± 0.3 ^b^**	**4.3 ± 0.3**	**6.76**	Verdim D	4.2 ± 0.3 ^a^	4.2 ± 0.0 ^a^	3.8 ± 0.2 ^a^	4.1 ± 0.3	6.17
Z39	3.7 ± 0.1 ^a^	3.8 ± 0.1 ^a^	3.3 ± 0.2 ^b^	3.6 ± 0.2	6.32	Emcapa 143	4.0 ± 0.2 ^a^	4.0 ± 0.1 ^a^	3.6 ± 0.2 ^b^	3.9 ± 0.2	5.53
Z35	4.2 ± 0.2 ^a^	4.2 ± 0.2 ^a^	3.9 ± 0.3 ^a^	4.1 ± 0.2	4.26	Ouro negro 1	3.9 ± 0.2 ^a^	3.5 ± 0.0 ^a^	3.5 ± 0.5 ^a^	3.6 ± 0.3	6.86
Z40	4.0 ± 0.1 ^a^	4.1 ± 0.1 ^a^	3.7 ± 0.1 ^b^	3.9 ± 0.2	5.29	Ouro negro 2	3.7 ± 0.2 ^b^	3.8 ± 0.0 ^b^	4.1 ± 0.1 ^a^	3.9 ± 0.2	5.51
Z29	4.2 ± 0.1 ^a^	4.0 ± 0.1 ^a^	3.6 ± 0.1 ^b^	3.9 ± 0.3	6.87	Clementino	3.9 ± 0.2 ^a^	3.9 ± 0.0 ^a^	3.6 ± 0.1 ^b^	3.8 ± 0.2	4.82

Note: * Average soluble solids content considering the three consecutive crops (2018, 2019, and 2020). CV: coefficient of variation. Different letters for the same genotype indicate statistical differences between crops by ANOVA (*p* < 0.05). Values in bold indicate genotypes with outstanding contents of soluble solids (>4 °Brix, CV < 7% in all three consecutive crops).

## Data Availability

Data supporting the reported results are available upon request.
